# Prebiotics from Seaweeds: An Ocean of Opportunity?

**DOI:** 10.3390/md17060327

**Published:** 2019-06-01

**Authors:** Paul Cherry, Supriya Yadav, Conall R. Strain, Philip J. Allsopp, Emeir M. McSorley, R. Paul Ross, Catherine Stanton

**Affiliations:** 1 Nutrition Innovation Centre for Food and Health, Ulster University, Cromore Road, Coleraine, Co. Londonderry BT52 1SA, UK; paul.cherry@ucc.ie (P.C.); pj.allsopp@ulster.ac.uk (P.J.A.); em.mcsorley@ulster.ac.uk (E.M.M.); 2 Teagasc Food Research Centre, Moorepark, Fermoy, P61 C996 Co. Cork, Ireland; 18supriya@gmail.com (S.Y.); conall.strain@teagasc.ie (C.R.S.); 3 APC Microbiome Ireland, University College Cork, T12 YT20 Cork, Ireland; p.ross@ucc.ie; 4 College of Science, Engineering and Food Science, University College Cork, T12 K8AF Cork, Ireland

**Keywords:** seaweed, gut microbiota, prebiotics, dietary fibre, complex polysaccharides, polyphenols, polyunsaturated fatty acids, carotenoids, phytochemicals

## Abstract

Seaweeds are an underexploited and potentially sustainable crop which offer a rich source of bioactive compounds, including novel complex polysaccharides, polyphenols, fatty acids, and carotenoids. The purported efficacies of these phytochemicals have led to potential functional food and nutraceutical applications which aim to protect against cardiometabolic and inflammatory risk factors associated with non-communicable diseases, such as obesity, type 2 diabetes, metabolic syndrome, cardiovascular disease, inflammatory bowel disease, and some cancers. Concurrent understanding that perturbations of gut microbial composition and metabolic function manifest throughout health and disease has led to dietary strategies, such as prebiotics, which exploit the diet-host-microbe paradigm to modulate the gut microbiota, such that host health is maintained or improved. The prebiotic definition was recently updated to “a substrate that is selectively utilised by host microorganisms conferring a health benefit”, which, given that previous discussion regarding seaweed prebiotics has focused upon saccharolytic fermentation, an opportunity is presented to explore how non-complex polysaccharide components from seaweeds may be metabolised by host microbial populations to benefit host health. Thus, this review provides an innovative approach to consider how the gut microbiota may utilise seaweed phytochemicals, such as polyphenols, polyunsaturated fatty acids, and carotenoids, and provides an updated discussion regarding the catabolism of seaweed-derived complex polysaccharides with potential prebiotic activity. Additional in vitro screening studies and in vivo animal studies are needed to identify potential prebiotics from seaweeds, alongside untargeted metabolomics to decipher microbial-derived metabolites from seaweeds. Furthermore, controlled human intervention studies with health-related end points to elucidate prebiotic efficacy are required.

## 1. Introduction

Seaweeds are an underexploited and sustainable crop which offer a rich source of bioactive compounds, including novel dietary fibres, polyphenols, fatty acids, and carotenoids [1,2]. Epidemiological evidence comparing Japanese and Western diets have correlated seaweed consumption (5.3 g/day in Japan) with decreased incidence of chronic disease [3], while the purported efficacies of seaweed phytochemicals have led to potential functional food and nutraceutical applications which aim to protect against cardiometabolic and inflammatory risk factors associated with non-communicable diseases, such as obesity, type two diabetes, metabolic syndrome, cardiovascular disease, inflammatory bowel disease, and some cancers [1]. 

Current understanding of mutualistic diet-host-microbe interactions has generated efforts to exploit diet to maintain health status, and to prevent or overcome non-communicable diseases, where an imbalance of gut microbiota composition and metabolic function manifests during the onset and pathophysiology of gastrointestinal, neurological, and cardio-metabolic diseases, often congruent with intestinal inflammation and compromised gut barrier function [4,5]. As such, it has become pertinent to explore dietary strategies which modulate gut microbial composition and function to improve host health. This includes the use of prebiotics as fermentable substrates to enable selective gut commensal metabolism. 

The prebiotic definition was recently updated to “a substrate that is selectively utilised by host microorganisms conferring a health benefit” [6], which includes the inhibition of pathogens, immune system activation, and vitamin synthesis and provides opportunity to explore the prebiotic efficacy of non-complex polysaccharide components such polyphenols, phytochemicals, and polyunsaturated fatty acids (PUFAs) [6]. It is also recognised that other microbial species have the potential to catabolise prebiotics, besides the classical examples of *Bifidobacterium* and *Lactobacillus* [6], courtesy of culture-independent techniques, such as 16S rRNA next generation sequencing and whole genome shotgun metagenomic sequencing which have provided taxonomic classification to identify microbial abundance/diversity and inferred or identified metabolic function [7].

Given that previous discussion regarding the prebiotic potential of seaweed components has focused solely upon the saccharolytic fermentation of complex polysaccharides and the physiological effects of short chain fatty acid metabolites (SCFAs) [3,8,9], scope exists to explore the prebiotic potential of other phytochemical components derived from seaweeds, namely polyphenols, carotenoids, and PUFAs, applicable to both human and animal health. 

This review aims to provide an updated discussion regarding the fermentation and potential prebiotic effect of seaweed polysaccharides and oligosaccharides, based on recent evidence from in vitro fermentation studies and in vivo animal models, and to postulate how other seaweed phytochemicals, such as polyphenols, PUFAs, and carotenoids, may interact with the gut microbiota to manipulate microbial composition and/or function to elicit bioactivities pertained to a prebiotic. The latter provides new opportunities to complete prebiotic screening studies using in vitro techniques and pre-clinical animal models to understand how parent compound biotransformation into endogenously-derived or gut microbiota-derived metabolites impact bioaccessibility and bioavailability to influence gut microbial community structure and function, conducive to a prebiotic effect. Evidence from clinical trials with health-related end-points and mechanistic insight is imperative to substantiate health claims associated with a prebiotic effect.

## 2. Complex Polysaccharides

Seaweeds contain 2.97–71.4% complex polysaccharides [2,3], which include alginate, fucoidan, and laminarin in brown seaweeds; xylan and sulphated galactans, such as agar, carrageenan, and porphyran in red seaweeds; whilst ulvan and xylan are found in green seaweeds. The monosaccharide composition of the major brown, red, and green seaweed glycans are presented in Table 1, Table 2, Table 3, respectively. Whilst no human study to date has explored prebiotic sources from seaweeds, several in vitro studies [10,11,12,13], and in vivo animal studies [14,15], have explored the prebiotic potential seaweeds and their polysaccharide components.

Seaweed polysaccharides are atypical in structure to terrestrial glycans, and are understood to resist gastric acidity, host digestive enzymes, and gastrointestinal absorption [8]. Seaweed glycans may, therefore, serve as fermentation substrates for specific gut microbial populations or facilitate substrate cross-feeding of partially broken-down intermediates, such as oligosaccharides and metabolic cross-feeding of SCFAs to cause indirect proliferation of specific bacteria [16,17,18,19,20]. The physiological effects of SCFAs, primarily acetate, propionate, and butyrate, include the reduction of luminal pH to inhibit pathogens, the provision of energy sources to colonocytes, and the activation of free fatty acid receptors; where acetate and propionate are ligands for anorexigenic pathways in appetite regulation and can inhibit the rate limiting step of hepatic cholesterol synthesis via 3-hydroxy-3-methylglutaryl CoA reductase inhibition [21,22,23].

To facilitate saccharolytic fermentation in the colon, the gut microbiota must express functional carbohydrate active enzymes (CAZymes) to catabolise seaweed glycans as carbon sources within the colonic digesta. The repertoire of CAZymes expressed by the human gut microbiota includes glycoside hydrolase and polysaccharide lyase families to facilitate degradation via hydrolysis and elimination reactions, respectively [24,25,26]. Whole genome sequencing has previously identified gene clusters which encode the catabolic machinery responsible for the breakdown of prebiotics, which includes the CAZyme families responsible for the catabolism of inulin, lactulose, fructo-oligosaccharides, xylo-oligosaccharides, and galacto-oligosaccharides by human gut commensal strains, including *Bifidobacterium longum* NCC2705, *Bifidobacterium adolescentis* ATCC 15703, *Streptococcus thermophilus* LMD9, *Eubacterium rectale* ATCC 33656, *Bacteroides vulgatus* ATCC 8482, and *Fecalibacterium prausnitzii* KLE1255 [27].

Based on open source data from the Carbohydrate-Active enZYmes Database [28], Table 1, Table 2 and Table 3 detail the CAZyme families which may exert specificity for seaweed glycans and highlights the gut bacterial populations which have demonstrable evidence for seaweed glycan utilisation. This is dominated by *Bacteroides*, which have extensive glycolytic versatility [26,29]. This may explain why in vitro batch culture fermentation data of seaweeds and seaweed glycans indicate the proliferation of *Bacteroides*; whilst the degradation of complex seaweed glycans by *Bacteroides* could also facilitate the cross-feeding of oligosaccharides, monosaccharides, and SCFAs for gut commensals deemed beneficial to health, including *Bifidobacterium*.

In vitro fermentation studies are frequently used as screening tools to model colonic fermentation and determine substrate utilisation by an ex vivo faecal inoculum, with seaweed as a sole carbon source. An overview of recent in vitro fermentation studies which have evaluated the fermentation of whole seaweeds or extracted complex polysaccharide components by the human gut microbiota is presented in Table 4 (brown seaweeds), Table 5 (red seaweeds), and Table 6 (green seaweeds). These tables include differences in study methodologies, for example, test substrate dosage; the use of an in vitro digestion before the fermentation experiment (declared within the methods section of the cited research paper); how the inoculum was prepared; duration of the faecal fermentation experiment; microbial enumeration method; and the analytical technique used to ascertain metabolite changes during the fermentation. The use of an in vitro digestion before in vitro fermentation is often used to determine whether a substrate is resistant to endogenous digestive enzymes and small intestinal absorption, and to provide the fraction of a dietary component which is bioaccessible in the colon [30]. The lack of an in vitro digestion before fermentation experiments may cause false positive results, given that low molecular weight components present in seaweed extracts, normally absorbed in the small intestine, are used as fermentation substrates for the ex vivo microbiota. Table 7 highlights data from in vivo rodent studies which have evaluated the potential prebiotic effect of seaweeds and seaweed glycans. 

### 2.1. Brown Seaweed Polysaccharides

Brown seaweeds are commonly used as food ingredients owing to their commercial abundance [53]. The anti-obesogenic effects of brown seaweeds are reported in mice, where supplementation of 5% (*w*/*w*) *Saccorhiza polyschides* extract, containing 12% dietary fibre, reduced body weight gain and fat mass of mice with diet-induced obesity [54]. The anti-obesogenic effect was attributed to the fermentation of alginate and fucoidan complex polysaccharide components, owing to reduced microbial bile salt hydrolase activity; however, no gut microbial compositional data were provided. Elsewhere, the in vitro evidence (Table 4) indicates that whole brown seaweeds and their extracted complex polysaccharide components are fermented by the ex vivo faecal microbiota, with increased production of acetate, propionate, butyrate, and total SCFAs reported during fermentation experiments. A corresponding increase in populations, such as *Bifidobacterium, Bacteroides*, *Lactobacillus, Roseburia*, *Parasutterella, Fusicatenibacter*, *Coprococcus, Fecalibacterium* is also reported [55,56,57]. 

#### 2.1.1. Alginate

Alginates are composed of 1,4-linked α-l-guluronic (G) and β-d-mannuronic acid (M) residues to form GM, GG and MM blocks, and represent 17−45% dry weight of brown seaweeds [58]. The colloidal properties of alginates have wide application in food processing, biotechnology, medicine and pharmaceutical industries [59], while degraded sodium alginate is an approved item of “foods with specified uses”, under the categories of “Foods that act on cholesterol plus gastrointestinal conditions” and “Foods that act on blood cholesterol levels” in Japan [60]. The presence of water soluble alginate oligosaccharides in the faeces of pigs fed alginate is indicative of alginate lyase activity by the luminal or mucus adherent gut microbiota [61], although an adaptation period of > 39 days is reported for the degradation of G blocks by the porcine microbiota whilst M blocks are readily degraded [62].

The capacity for alginate to modulate the gut microbiota of Japanese individuals was highlighted over 20 years ago [63], where alginate supplementation (30 kDa, 10 g/day, n = 8) significantly increased faecal *Bifidobacterium* populations in healthy male volunteers after both one and two weeks, alongside significantly increased acetic and propionic acids after two weeks. Deleterious metabolites, including faecal sulphide, phenol, p-cresol, indole, ammonia and skatole were significantly reduced compared to the control (free living) diet. Notably, faecal *Bifidobacterium* counts and SCFA concentrations returned to baseline in the week after alginate diet cessation, which highlights the transient nature of the gut microbiota and the need for greater powered long-term human intervention studies.

Subsequent in vitro fermentation studies have indicated that alginate is fermented by the human gut microbiota, for example, a 24 h in vitro fermentation of a 212 kDa alginate increased total bacterial populations, although no statistical increase in individual *Bifidobacterium*, *Bacteroides*/*Prevotella*, *Lactobacillus*/*Enterococcus*, *Eubacterium rectale*/*Clostridium coccoides*, or *Clostridium histolyticum* populations were observed [11]. Acetic acid, propionic acid and total SCFAs were significantly increased after 24 h fermentation with the 212 kDa alginate, while alginate of 97 kDa increased total SCFA and acetate production after 10 h of fermentation. Alginates of 38 kDa, and 97 kDa did not change microbial abundance, although the authors could not correlate molecular weight with fermentation patterns.

Alginate oligosaccharides (AOS) (~3.5 kDa) can be obtained via acidic or enzymatic hydrolysis of alginate polysaccharides [58], and enzymatically derived AOS has promoted the growth of *Bifidobacterium bifidum* ATCC 29521, *Bifidobacterium longum* SMU 27001 and *Lactobacilli*, in vitro [64,65]. Supplementation of 2.5% AOS for two weeks significantly increased faecal *Bifidobacterium*. in rats compared to control and 5% FOS supplemented diets (13-fold and 4.7-fold increase, respectively), while faecal *Lactobacillus* were 5-fold greater in rats who consumed AOS compared to FOS. *Enterobacteriaceae* and *Enterococcus* populations were significantly decreased following AOS supplementation. Elsewhere, the hydrolysis of alginate, mannuronic acid oligosaccharides (MO) and guluronic oligosaccharides (GO) during a 48 h batch culture fermentation with the faecal microbiota of Chinese individuals demonstrated increased production of acetate, propionate, butyrate, and total SCFAs compared to the substrate-free control, where GO generated the greatest increase [36]. Subsequent strain isolation from the stools of individuals who demonstrated alginate degradation during fermentation identified *Bacteroides xylanisovlens* G25, *Bacteroides thetaiotomicron* A12, *Bacteroides ovatus* A9, and *Bacteroides ovatus* G19 as strains capable of hydrolysing alginate and AOS, where *Bacteroides ovatus* G19 expressed α-1,4-guluronanlyase and β-1,4-mannuronanlyase CAZymes [34]. 

A *Bacteroides xylanisolvens* strain with 99% similarity to *Bacteroides xylanisolvens* XB1A was recently isolated from the stool of a Chinese individual and the alginate lyase gene expressed was 100% homologous to the alginate lyase of *Bacteroides ovatus* strain ATCC 8483 [33]. The preceding in vitro fermentation study demonstrated increased production of acetate, propionate, butyrate, and total SCFAs compared to the soluble starch control vessel following a 72 h fermentation of alginate. 

Alginate lyase depolymerises alginate polysaccharides to lower molecular weight oligosaccharides via β-elimination, and is most commonly expressed by marine bacteria, including *Flammeovirga*, *Vibrio*, *Pseudoalteromonas*, *Glaciecola chathamensis* S18K6, and *Zobellia galactanivorans* [65,66,67,68,69], while the terrestrial bacteria *Paenibacillus* sp. Strain MY03 was recently reported to possess genes encoding alginate lyase and agarase enzymes [70]. The acquisition of genes encoding alginate lyase enzymes by human gut *Bacteroides* is a suggested consequence of horizontal gene transfer from the marine environment [31,71], where seaweed consumption may have provided a vector to exert a selective pressure to induce diet-driven adaptations of the gut microbiota [35,72,73,74,75,76]. Recent work by Matthieu et al. [35] suggests that an alginate degradation system within the genome of human gut *Bacteroides* was a result of ancient acquisition, where the polysaccharide utilisation loci encodes PL6 and PL17 alginate lyase enzymes and hypothetical proteins responsible for alginate recognition, internalisation, and catabolism, including bacterial ABC transporter proteins to facilitate alginate uptake across the bacterial membrane [77]. Nevertheless, in vivo rodent studies have demonstrated that seaweed glycans are fermented even though animals have never been exposed to dietary seaweeds before the intervention, which suggests that the gut microbiome contains genes for CAZymes which can degrade seaweed glycans when expressed.

#### 2.1.2. Laminarin

Laminarin is a water-soluble storage polysaccharide consisting of 1,3- or 1,6-β-glucose with an average molecular weight of 5 kDa [78] and accounts for 10–35% of the dry weight of brown seaweeds [58]. One in vitro batch culture fermentation of laminarin demonstrated increased *Bifidobacteria* and *Bacteroides* after 24 h [79], while another demonstrated increased propionate and butyrate production after 24 h [14]. A subsequent in vivo rat study (143 mg laminarin per kg body weight per day for 14 days) indicated that laminarin was not selectively fermented by *Lactobacillus* and *Bifidobacterium*, but could modify jejunal, ileal, caecal and colonic mucus composition, secretion, and metabolism to protect against bacterial translocation. The authors suggest that increased luminal acidity and/or catabolism of laminarin by mucolytic commensals could elicit such effects, which corroborates the evidence that a complex polysaccharide-rich diet maintains mucus layer integrity to promote gut barrier function [80,81]. Future studies regarding intestinal mucus modulation by laminarin may wish to characterise gut microbiota compositional and functional changes following laminarin ingestion, to detect the abundance and metabolic activity of glycan degraders, such as *Bacteroides* [82,83] or mucolytic species associated with health, such as *Akkermansia muciniphila* or *Ruminococcus* [84,85]. Elsewhere, laminarin increased L-cell GLP-1 secretion to attenuate diet-induced obesity in mice, and improved glucose homeostasis and insulin sensitivity [86]. The authors suggested that the observed cytosolic Ca^2+^ cascade caused GLP-1 secretion, which is in agreement with GPR41/43 receptor activation by SCFAs produced by gut microbial fermentation [87,88], however, data obtained to assess laminarin-induced changes to gut microbiota composition and metabolic output is needed to ascribe a prebiotic effect in this study. 

The abundance of glycoside hydrolase and β-glucosidase enzymes expressed by the human gut microbiota may have the capacity to catabolise laminarin [24,89,90,91], for example, a *Bacteroides cellulosyliticus* WH2 human gut isolate was able to grow on laminarin-supplemented minimal media in vitro, (incidentally it did not grow on alginate, carrageenan, or porphyran) [92]; however, the molecular mechanisms by which human gut *Bacteroides* breakdown laminarin are likely distinct from those responsible for the degradation of mix linked β 1,3- 1,4- glucans, such as those found in cereals (e.g., by BoGH16_MLG_) [93].

#### 2.1.3. Fucoidan

Fucoidans are water soluble polysaccharides composed of sulphated 1,2- or 1,3- or 1,4-α-l-fucose which exist as structural polysaccharides in brown seaweeds and occupy 5–20% of algal dry weight [58,94]. The structural heterogeneity of fucoidan encompasses varying degrees of branching, sulphate content, polydispersity, and irregular monomer patterns, which can include fucose, uronic acid, galactose, xylose, arabinose, mannose, and glucose residues [9,59,95]. 

A recent in vitro fermentation study of fucoidan (<30 kDa) extracted from *Laminaria japonica* demonstrated a greater increase in *Bifidobacterium* and *Lactobacillus* following 24 h and 48 h fermentation relative to >30 kDa fucoidan [12], while fucoidan from *Ascophyllum nodosum* (1330 kDa) and *Laminaria japonica* (310 kDa) were shown to increase *Lactobacillus* and *Ruminococcaceae*, respectively, in the caecal microbiota of mice gavaged with 100 mg/kg/day [96]. Fucoidan also reduced serum LPS-binding protein levels in this study—indicative of a reduced antigen load and reduced inflammatory response. In contrast, fucoidan with a fucose-rich and highly sulphated fucoidan extracted from *Cladosiphon okamuranus* was not fermented by the rat gut microbiota [97]. 

While the purported bioactivities of fucoidan include anti-obesogenic, anti-diabetic, anti-microbial, and anti-cancer properties [98], there is limited evidence to implicate a role for the gut microbiota with such bioactivities, and studies are needed to evaluate the structure-dependent fermentation of fucoidan to ascribe a prebiotic effect. For the latter, this is surprising given the myriad of α-fucosidase enzymes present in the human gut bacterial glycobiome.

### 2.2. Red Seaweed Polysaccharides

#### 2.2.1. Galactans (Carrageenan, Agar, and Porphyran)

Red seaweeds, such as *Gelidium* spp. and *Gracilaria* spp., are used in the commercial production of agar and carrageenan food additives, including thickening, stabilizing and encapsulation agents [53]. A summary of evidence from recent in vitro fermentation experiments using red seaweed-derived substrates are presented in Table 5.

Carrageenans are composed of sulphated 1,4-β-d-galactose, 1,3-α-d-galactose, and 3,6-anhydro-d-galactose [43], and constitutes 30−75% dry weight of red seaweeds [58]. In rats fed 2.5% *Chondrus crispus*, of which carrageenan is a major polysaccharide component, faecal *Bifidobacterium breve*, and acetate, propionate, and butyrate SCFAs were significantly increased alongside a significant decrease in the pathogens *Clostridium septicum* and *Streptococcus pneumonia*, as compared to the basal diet [15]. Furthermore, a 1:1 mixture of polysaccharide extracts from *Kappaphycus alvarezii* (containing carrageenan) and *Sargassum polycystum* (brown seaweed) has lowered serum lipids in rats [39]. In a study by Li et al. [34], β-carrageenase activity in a *Bacteroides uniforms* 38F6 isolate complex of *Bacteroides xylanisolvens* and *Escherichia coli* hydrolysed κ-carrageenan oligosaccharides into 4-*O*-sulfate-d-galactose, κ-carratriose, κ-carrapentaose, and κ-carraheptaose, which could facilitate cross-feeding to promote the growth of *Bifidobacterium* populations. 

Agar is composed of sulphated 1,3-β-d-galactose and 1,4- 3,6-anhydro-α-l-galactose [40] and can be fractionated into agarose and agaropectin [8]. Low molecular weight agar of 64.64 kDa has demonstrated a bifidogenic effect alongside increased acetate and propionate SCFA concentrations after 24 h in vitro fermentation with human stool inoculum [11], while mice fed with 2.5% (*w*/*v*) neoagarose oligosaccharides for 7 days demonstrated increased caecal and faecal *Lactobacillus* and *Bifidobacterium* [102]. The utilisation of agaro-oligosaccharides was noted in vitro by *Bacteroides uniforms* L8, isolated from Chinese individuals, which secreted a β-agarase CAZyme to breakdown agarooligosaccharides into agarotriose and subsequently facilitated the growth of *Bifidobacterium infantis* and *Bifidobacterium adolescentis* via the cross feeding of agarotriose [103]. 

Porphyran is made up of sulphated 1,3-β-d-galactose, 1,4-α-l-galactose-6-sulfate and 3,6-anhydro-α-l-galactose [41,104,105]. An in vitro faecal fermentation study indicated that porphyran did not significantly increase SCFAs, but stimulated *Lactobacillus* and *Bacteroides* populations [79]. While pure cultures of *Bifidobacterium breve, Bifidobacterium longum, Bifidobacterium infantis, Bifidobacterium adolescentis,* but not *Bifidobacterium bifidum*, were able to ferment dried *Porphyra yezoensis* (Nori), containing a low protein content (25%), whereas Nori with a high protein content (41%) was not fermented [105]. It is likely that carbohydrate content was highest in the low protein Nori, thus seasonal- and species-variation and in seaweed macronutrient content should be considered a determinant factor for the fermentability of whole seaweeds [106,107,108,109].

Evidence for the horizontal transfer of genes for porphyranase and agarase CAZymes from the marine bacteria, *Zobellia galactanivorans*, to *Bacteroides plebeius* of Japanese individuals is indicative of diet-driven adaptations of the human gut microbiome [41,44]; however, the North American counterparts in this study did not consume seaweeds and the gut microbiota of these individuals did not express such CAZymes. This may mean that the fermentation of seaweed polysaccharides, such as porphyran and agar, requires exposure to, and acquisition of, specific CAZymes usually present in the marine environment [73]. Red seaweed galactans are emerging prebiotic candidates given the commercial availability of red seaweed hydrocolloids and the potential gut modulatory effects of oligosaccharides obtained from red seaweeds. Nevertheless, further in vivo evidence is needed, given the purported pro-inflammatory effects of low molecular weight carrageenan [110,111,112]. 

#### 2.2.2. Xylan

Xylan, composed of 1,3-1,4-β-D-xylose, is a major constituent of red seaweeds, such as *Palmaria palmata* [46]. A previous in vitro faecal fermentation study of xylan derived from *P*. *palmata*, reported that xylose was fermented after six h alongside a 58:28:14 ratio of acetate, propionate, and butyrate SCFAs (total SCFAs were 107 mM/L) [113]. This study did not ascertain bacterial compositional data, and thus a knowledge gap is presented given that xylans and xylooligosaccharides (XOS) extracted from terrestrial plants, such as wheat husks and maize, are mooted as potential prebiotics owing to evidence of bifidogenesis, improved plasma lipid profile, and positive modulation of immune function markers in healthy adults [114,115]. Given that human gut *Bacteroides* express a repertoire of xylanase and xylosidase CAZymes [116], investigations regarding the capacity of the human gut microbiota to catabolise red seaweed xylans and XOS are suggested.

### 2.3. Green Seaweed Polysaccharides

#### Ulvan

Ulvans are water-soluble cell wall polysaccharides that account for 8–29% dry weight of green seaweeds, and are composed of sulphated 1,3-α-l-rhamnose, 1,4-β-d-glucuronic acid, and 1,4-β-d-xyloglucan [51]. Previous reports indicate that *Ulva lactuca* and ulvan polysaccharides are poorly fermented by the human gut microbiota [8,95,118], while an in vitro fermentation study of *Enteromorpha* spp. With a human faecal inoculum reported no difference in *Enterococcus, Lactobacillus*, and *Bifidobacterium* populations compared to the control; only an increase in *Enterobacter* after 24 h and 48 h of fermentation (Table 6) [12]. In contrast, a recent in vitro faecal fermentation study indicated that Ulvan stimulated the growth of *Bifidobacterium* and *Lactobacillus* populations and promoted the production of lactate and acetate [79]. Further, a murine study showed that *Enteromorpha* (EP) and *Enteromorpha* polysaccharides (PEP) ameliorated inflammation associated with Loperamide-induced constipation in mice [119], where alpha diversity, Firmicutes, and Actinobacteria were increased in the faecal microbiota of seaweed-supplemented mice compared to the constipated control. Bacteroidetes and Proteobacteria were decreased, while Bacteroidales family *S24-7* and *Prevotellaceae* were increased in EP and PEP, respectively. Current evidence for the fermentation of green seaweeds and their polysaccharides is limited and fermentation may require specific α-l-rhamnosidase activity by gut commensals [50]. More experimental evidence is needed to understand the impact of ulvans and ulvan-oligosaccharides in the human and animal diet. 

### 2.4. Future Prospective–Obtaining Oligosaccharides

Enzyme technologies are reported to increase the extraction yield and reduce the molecular weight of bioactive components obtained from seaweeds, with examples of enhanced prebiotic activity when commercially available cellulases or seaweed-specific enzymes were used to hydrolyse polysaccharides [99,120]. Despite limited commercial availability of seaweed-specific enzymes, an avenue for functional oligosaccharide production is presented if efforts to develop commercially viable saccharolytic enzymes from microorganisms (primarily marine). Examples of such glycoside hydrolases include fucoidanase from *Sphingomonas paucimobilis* PF-1 [121]; ulvan lyase from *Alteromonas* spp. [122] and the family *Flavobacteriaceae* [123]; β-agarase from *Cellulophaga omnivescoria* W5C [124] and *Cellvibrio PR1* [125]; alginate lyase from *Flammeovirga* [126], and *Paenibacillus* [70]; and laminarinase from *Clostridiium thermocellum* [127]. Factors which influence the stability and efficacy of such hydrolytic enzymes may include metal ion interaction, or thermostability at the high temperatures needed to prevent gelling of polysaccharides. Recent insight into the production of agarose oligosaccharides and neoagarose oligosaccharides from agar exemplify this [128].

## 3. Polyphenols

Seaweeds are rich in polyphenols, such as catechins, flavonols, and phlorotannins. Red and green seaweeds are a source of bromophenols, phenolic acids, and flavonoids [132], while phlorotannins are the most abundant polyphenol in brown seaweeds. Most research to date concerns the bioactivity of phlorotannins, a class of polyphenol unique to brown algae comprised of phloroglucinol monomers and categorised as eckols, fucols, fuhalols, ishofuhalols, phloroethols, or fucophloroethols [132]. The purported bioactivities of seaweed polyphenols are associated with the mitigation of risk factors pertained to type 2 diabetes and cardiovascular disease, including hyperglycemia, hyperlipidemia, inflammation and oxidative stress [133,134,135,136,137], and also anti-microbial activity [138]. Owing to heterogeneity in both molecular weight and the level of isomerisation, characterisation of polyphenols is difficult [139,140,141], and a paucity of information exists regarding the endogenous digestion and microbial catabolism of seaweed polyphenols, with a scarce mechanistic understanding of how they may exert health benefits via the gut microbiota. 

Most polyphenols of plant origin must undergo intestinal biotransformation by endogenous enzymes and the gut microbiota prior to absorption across enterocytes. These enzymatic transformations include the elimination of glycosidic bonds, for example, flavonoids are converted to glycones (sugars) and aglycones (non-sugars–polyphenols) by endogenous β-glucosidases in the small intestine [142]. The transport of aglycones to the liver via the portal vein results in phase II biotransformation (coupling reactions, chiefly hepatic conjugation to O-glucuronides and O-sulfates) to facilitate urinary and biliary elimination. Phase II metabolites are absorbed into the systemic circulation, or excreted in bile and re-enter the duodenum (hepatic recycling), where subsequent glucuronidase, glycosidase, or sulphatase-mediated deconjugation by the colonic microbiota may favour aglycone reabsorption [143].

Approximately 90–95% of dietary polyphenols reach the colon intact [144], where biotransformation and metabolism by the gut microbiota occurs via hydrolysis, reduction, decarboxylation, demethylation, dehydroxylation, isomerisation, and fission [145], to produce low-molecular weight compounds with less chemical heterogeneity than the polyphenol parent compound [142]. It is suggested that a complex network of gut microbial species is necessary for full biotransformation of polyphenols, whereas simple reactions, such as deglycosylation, can be achieved by individual gut strains. Furthermore, the bioactivities associated with dietary polyphenol intake may be dependent on the catabolic capacity and composition of the gut microbiota, owing to the biological activity of metabolites rather than the parent polyphenol compound present in food [146,147], while a synergistic effect between prebiotic polyphenols and probiotic bacteria may occur [6].

The identification of bacteria which possess the metabolic capabilities to utilise polyphenols was previously identified in *Eubacterium oxidoreducens*, which could catabolise gallate, pyrogallol, phloroglucinol and quercetin [148]. Quercetin biotransformation by *Eubacterium ramulus* has also been identified [149], and multiple human gut microbes which possess phenolic enzymes capable of breaking down glycosides, glucuronides, sulphates, esters, and lactones was summarised by Selma et al. [145]. Such microorganisms included *E. coli* with β-glucuronidase activity; *Eubacterium*, *Bacteroides*, and *Clostridium* with β-glucosidase activity; *Lactobacillus*, *Eubacterium*, *Clostridium* , *Butyrbacterium*, *Streptococcus*, and *Methylotrophicum* with demethylase activity; and *E. coli*, *Bifidobacterium*, *Lactobacillus*, *Bacteroides*, *Streptococcus* , *Ruminococcus*, and *Enterococcus* with esterase activity. There is also evidence for α-l-Rhamnosidase mediated hydrolysis of rutinose, present on glycosylated polyphenols (rhamnoglycosides), to produce aglycones, by species, such as *Bacteroides thetaiotaomicron* [50], *Bifidobacterium dentium* [150], *Bifidobacterium catenulatum* [151], *Bifidobacterium pseudocatenulatum* [151], and *Lactobacillus plantarum* [152].

Current knowledge regarding the fate of seaweed polyphenols in the human gastrointestinal tract is scarce; however, it is understood that the limited absorption of *Ascophyllum nodosum* polyphenols from small intestinal enterocytes to the portal vein may facilitate the conjugation of polyphenols to methylated, glucuronidated, or sulphated forms rather than hydrolysis to aglycones [153,154]. Subsequently, unabsorbed conjugated polyphenols are available for biotransformation by the colonic microbiota, then potentially absorbed across the colonocytes. Indeed, Corona et al. [154], observed a reduction of total polyphenol contents of an *Ascophyllum nodosum* polyphenol extract, high molecular weight fraction (>10 kDa), and low molecular weight fraction (1–10 kDa) following in vitro digestion and batch culture fermentation; although anti-genotoxic activity against H_2_O_2_ induced DNA damage of HT-29 cells was increased (to a greater extent by the high molecular weight fraction). This study did not assess the microbiota composition, however, elsewhere, an in vitro fermentation of an *Ecklonia radiata* phlorotannin extract significantly increased *Bacteroidetes*, *Clostridium coccoides*, *E. coli*, and *Fecalibacterium prausnitzii*, but decreased *Bifidobacterium* and *Lactobacillus* populations after 24 h fermentation [56]. More in vitro digestion studies would be useful to understand the stability of seaweed polyphenols as extracts or within the seaweed matrix [155,156]. These studies may be complemented by studies which use ileostomy patient cohorts to determine structural changes to seaweed polyphenols following upper GI digestion in vivo to indicate polyphenol bioaccessibility in the colon [157]. 

A recent review highlighted the potential for dietary polyphenols to modulate the gut microbiota by increasing *Bifidobacterium, Lactobacillus, Bacteroides*, *Enterococcus, Akkermansia muciniphila*, and *Fecalibacterium prausnitzii* populations [158]. This review did not include any studies which assessed modulation of the gut microbiota by seaweed polyphenols and, therefore, a research opportunity is presented. Inter-individual variation of gut microbiota composition and function is the key determinant for gut microbiota-mediated biotransformation of phenolic compounds to bioactive metabolites [159,160]. Therefore, identification of bacterial species or strains with the ability to catabolise seaweed polyphenols and their respective catabolic machinery is needed to understand if seaweed polyphenols could be prebiotic [161,162]. Moreover, considering that gut microbiota-derived secondary metabolites reach a peak plasma concentration much later than the original aglycone or hepatic conjugates, controlled nutrikinetic studies could elude how dietary polyphenols from seaweeds interact with host-microbiota metabolism [163,164]. Identification of faecal, urinary, serum, or tissue biomarkers via untargeted and targeted metabolomics approaches, and the use of stable isotope studies, may also indicate variation in synthesis, bioavailability, metabolism, and excretion of polyphenols and associated metabolites. While integration of dose response studies alongside metagenomics and metabolomics analyses, akin to those conducted for berry and wine polyphenols, could elude how much seaweed polyphenol is required to have an impact, if any, on gut microbial composition, metabolic function, and host health [165,166].

## 4. Other Seaweed Phytochemicals

### 4.1. Carotenoids

Carotenoids are lipid soluble compounds which function within the photosynthetic machinery of seaweeds to produce pigments. Fucoxanthin is the predominant carotenoid in brown seaweeds [167], while lutein, β-carotene, astaxanthin, echinenone, violaxanthin, neoxanthin, and zeaxanthin are found in red and green seaweeds. Carotenoids are used as food colouring additives, while the application of fucoxanthin as functional food ingredients is suggested, owing to putative anti-oxidant, anti-inflammatory, anti-cancer, anti-obesity, and anti-diabetic bioactivities [168,169,170,171,172,173].

While some carotenoids are absorbed by enterocytes and converted into vitamin A and retinoid derivatives by endogenous beta-carotene oxygenase 1 (BCO1) and beta-carotene dioxygenase 2 (BCO2) enzymes [174,175], the bioavailability of carotenoids in the blood is reported as 10−40% [176], which has led to suggestions that carotenoids could be fermented by the gut microbiota [174,177]. The only evidence to date has demonstrated that male C57BL/6J mice supplemented with 0.04% (*w*/*w*) astaxanthin during an eight-week pilot study had increased abundance of caecal *Bifidobacterium* [178], whereas *Proteobacteria* and *Bacteroides* were significantly increased in the caecum of BCO2 knockout mice; however, analysis of health biomarkers was not reported. Given the differences in microbiota composition between wild type and BCO2 knockout mice in this study, there is scope to investigate how carotenoids and their endogenous derivatives interact with the gut microbiota. Looking ahead, the use of in vitro models of gastrointestinal digestion and colonic fermentation would be useful to assess whether there is a direct substrate to microbiota effect or a host–microbe effect [179].

### 4.2. Polyunsaturated Fatty Acids (PUFAs)

The lipid content of seaweed ranges from 1–5% dry weight, which includes n-3 PUFAs, such as eicosapentanoic acid (EPA) and docosahexaenoic acid (DHA) [180,181]. The n-3 PUFA are associated with the anti-inflammatory activity to reduce cardiovascular disease risk and may also exert beneficial effects on brain function and behaviour, as mediated by the microbiota-gut-brain axis [182]. Dietary EPA and DHA intake are reported to improve microbial diversity, reduce the Firmicutes/Bacteroidetes ratio, reduce LPS-producing bacteria, and increase populations of *Bifidobacterium*, *Lachnospiraceae*, and lipopolysaccharide (LPS)-suppressing bacteria in both humans and animal models [182,183,184]. Although the evidence to date has focused on fish-derived n-3 PUFA, great scope exists to evaluate the prebiotic effect of n-3 PUFA obtained from seaweeds.

## 5. Fermented Foods

Fermented foods are understood to have improved nutritional and functional properties owing to bioactive or bioavailable components [185]. Seaweeds (mainly kelp) are a common vegetable ingredient in the fermented food, Kimchi. The microbial content of kimchi provides a source of probiotics, nutrients, and bioactive metabolites, which are reported to have anti-microbial, anti-oxidant, and anti-obesogenic activities [186,187,188]. One randomised controlled trial (RCT) observed that consumption of a seaweed Kimchi made from *L. japonica* for four weeks promoted the growth and survival of gut microbial lactic acid bacteria in humans [189], whilst another RCT concluded that consumption of 1.5 g/day fermented *L. japonica* containing 5.56% γ-aminobutyric acid (GABA) (*Lactobacillus brevis* BJ2 culture) was associated with a reduction in oxidative stress in healthy adults over four weeks, indicated by decreased serum γ-glutamyltransferse (GGT) and malondialdehyde, and increased antioxidant activity of superoxide dismutase and catalase compared to the placebo [190]. The latter study indicates that foods containing fermented brown seaweeds, such as *L. japonica*, may offer a novel source of GABA enriched ingredients, which are associated with hypotensive and anti-inflammatory effects [188]. Anti-oxidant, anti-diabetic, and anti-hypertensive efficacies are also reported for Korean rice wine fermented with *L. japonica* [191], while *Sargassum* fermented with a starter culture of *Enterococcus faecium* was reported to contain higher soluble polyphenol and mannuronic acid-rich alginate contents [192], which may increase the provision of microbiota accessible components for colonic fermentation. 

Reports of the functional properties of fermented foods containing red seaweeds are scarce; however, examples of red seaweed fermented foods include a fermented *Porphyra yezoensis* seaweed sauce, which used the marine halophilic lactic acid bacteria, *Tetragenococcus halophilus*, as a starter culture [193]; a *Gracilaria domingensis* aqueous extract applied as a texture modifier in fermented milks as a non-animal alternative to gelatin [194]; and carrageenan as a salt replacer in the production of fat-free cheese [195]. 

Given the availability of red, brown, and green seaweeds both commercially and locally [196], the production of seaweed-containing fermented foods could be a cost-effective alternative to bioactive component extraction. Nevertheless, an understanding of how live bacteria and bacterial metabolites present in fermented foods contribute towards health is required [185].

## 6. Seaweeds and Animal Health

Seaweeds also have a historical use as animal feed ingredients [197]. The capacity for seaweeds to modulate the gut microbiota of monogastrics, such as pigs and hens, is presented in Table 8 and Table 9, respectively, which complements the recent evidence for the application of seaweed bioactives in monogastric animal feed [198]. Table 8 shows limited evidence that the β-glucan, laminarin, may increase *Lactobacillus* populations but not *Bifidobacterium* populations. While there is scarce evidence for the selective stimulation of health-associated bacteria in pigs by the sulphated fucose, fucoidan. Only one recent study has evaluated the effect of dietary alginate on the porcine microbiota, where the genera *Ruminococcus*, *Roseburia* and *Lachnospira*, and an unclassified bacterium of the *F16* family were increased, alongside a significant decrease in the genus *Blautia,* the family *Clostridiaceae*, and an unclassified bacterium of *RF39* family [199]. In Table 9, recent evidence indicates that hens fed *Chondrus crispus* and *Sarcodiotheca gaudichaudii* red seaweeds may increase ceacal SCFA concentrations and modulate populations of *Bifidobacterium longum*, *Lactobacillus acidophilus*, *Streptococcus salivarius*, and *Clostridium perfringens* [200,201]; however, a bidirectional change in microbial composition was dose dependent and has only been assessed in two studies to date. Given the use of pigs as an animal model of humans [202], data from in vivo monogastric studies which are designed to evaluate the prebiotic potential of dietary seaweeds and seaweed-derived components could provide insight into the potential for human applications.

Table 10 and Table 11 summarise recent studies which have examined the impact of seaweed diets on the ruminant microbiota of cows and sheep, respectively, with the potential application of reducing methane production. Despite demonstrating decreased methane production, the cow rumen in vitro fermentation studies presented in Table 10 did not assess microbiota compositional changes, thus a knowledge gap is presented to understand which bacteria (if any), are increased or decreased, and are associated with a reduction in methane production. Table 11 shows that methanogenic bacteria and methane production were significantly decreased compared to the basal grass substrate control following the in vitro fermentation of sheep rumen with the red seaweed *Asparagopsis taxiformis* [203]. While sheep given an ad libitum diet of *Ascophyllum nodosum* brown seaweed (1%, 3%, or 5% *w*/*w*) for 21 days demonstrated a dose-dependent decrease in propionate and butyrate SCFAs and a dose-dependent increase in acetate [204], while several bacteria were significantly decreased, including *Prevotella copri*, *Roseburia,* and *Coprococcus*, while *Blautia producta* and the family *Veillonellaceae* were significantly increased compared to the basal diet. Moreover, the specific case of seaweed-fed North Ronaldsay sheep highlights how isolated organisms of the ruminant microbiome, such as *Prevotella*, *Clostridium butyricum*, *Butyrivibrio fibrisolvens*, and Spirochaetes have adapted to hydrolyse alginate laminarin, and fucoidan [205,206]. However, there is a paucity of evidence to implicate any health benefits attributed to a seaweed diet in these animals.

## 7. Conclusions

Current evidence regarding the prebiotic effects of seaweeds is dominated by complex polysaccharide components. This is because prebiotic research was previously focused on saccharolytic fermentation by the gut microbiota. Accumulating evidence from in vitro and in vivo animal studies provides encouraging data regarding the utilisation of red seaweed galactans and brown seaweed glycans, such as alginates and laminarins, with minor evidence for fucoidan and the green seaweed polysaccharide, ulvan. 

Given that the most recent definition of prebiotic places non-complex polysaccharide components in vogue, an opportunity is presented to explore how other seaweed phytochemicals, including polyphenols, carotenoids, and PUFAs, are metabolised by host microbial populations to benefit host health. Future investigations should consider the use of in vitro screening studies and in vivo animal studies to identify putative prebiotic compounds from seaweeds via the identification of host organisms which utilise seaweed components and the bioactive metabolites produced (via untargeted metabolomics). Furthermore, controlled human intervention studies with health-related end points to elucidate prebiotic efficacy are required.

## Figures and Tables

**Table 1:** Potential degradation of brown seaweed glycans by the human gut microbiota.

Carbohydrate		Carbohydrate-Active Enzyme (CAZyme)	Evidenced Glycolytic Bacteria	Reference
Alginate	1,4-β-d-mannuronic acid α-l-guluronic acid	PL6 Alginate lyase PL6 MG-specific alginate lyase	*Bacteroides clarus* *Bacteroides eggerthii*	
		PL15 Alginate lyase PL15 Oligoalginate lyase	*Bacteroides ovatus* *Bacteroides thetaiotaomicron* *Bacteroides xylanisolvens*	[31,32,33,34,35,36]
		PL17 Alginate lyase PL17 Oligoalginate lyase	*Bacteroides clarus* *Bacteroides eggerthii*	
Fucoidan	Sulphated 1,2-1,3-1,4-α-l-fucose	GH29 α-l-fucosidase GH29 α-1,3/1,4-l-fucosidase	Not determined	[37]
		GH95 α-l-fucosidase GH95 α-1,2-l-fucosidase		
Laminarin	1,3-1,6-β-glucose	GH16 β-glucanase GH16 β-1,3-1,4-glucanase GH16 endo-1,3-β-glucanase	*Bacteroides distasonis* *Bacteroides fragilis* *Bacteroides thetaiotaomicron*	[10,38]

PL, Polysaccharide Lyase family; GH, Glycoside Hydrolase family. Potential glycolytic bacteria were identified using the Carbohydrate-Active enZYmes Database [28].

**Table 2:** Potential degradation of red seaweed glycans by the human gut microbiota.

Carbohydrate		Carbohydrate-Active Enzyme (CAZyme)	Evidenced Glycolytic Bacteria	Reference
Agar (Galactan)	1,3-β-d-galactose 1,4-3,6-anhydro-α-l-galactose	GH2 β-galactosidase	*Bacteroidetes plebeius*	[39,40,41,42]
		GH16 β-agarase		
		GH86 β-agarase		
		GH117 1,3-α-3,6-anhydro-l-galactosidase		
Carrageenan (Galactan)	1,4-β-d-galactose 1,3-α-d-galactose 3,6-anhydro-d-galactose	GH2 β-galactosidase	*Bacteroides plebeius*	[41,43]
		GH117 1,3-α-3,6-anhydro-l-galactosidase		
Porphyran (Galactan)	Sulphated 1,3-β-d-galactose 1,4-α-l-galactose-6-sulfate 3,6-anhydro-α-l-galactose	GH16 β-porphyranase GH86 β-porphyranase	*Bacteroides plebeius*	[41,44,45]
Xylan	1,3-1,4-β-d-xylose	GH3 xylan 1,4-β-xylosidase	Not determined	[46,47,48,49]
		GH5 endo-1,4-β-xylanase		
		GH10 endo-1,4-β-xylanase		
		GH10 endo-1,3-β-xylanase		
		GH11 endo-β-1,4-xylanase		
		GH11 endo-β-1,3-xylanase		
		GH43 β-xylosidase		
		GH43 xylanase		
		GH43 β-1,3-xylosidase		
		GH67 xylan α-1,2-glucuronidase		
		GH115 xylan α-1,2-glucuronidase		
		CE1−CE7 and CE12 acetyl xylanesterases		

PL, Polysaccharide Lyase family; GH, Glycoside Hydrolase family. Potential glycolytic bacteria were identified using the Carbohydrate-Active enZYmes Database [28].

**Table 3:** Potential degradation of green seaweed glycans by the human gut microbiota.

Carbohydrate		Carbohydrate-Active Enzyme (CAZyme)	Evidenced Glycolytic Bacteria	Reference
Ulvan	Sulphated 1,4-β-d-Glucuronic acid α-l-Rhamnose 1,4-β-d-xyloglucan	GH78 α-l-rhamnosidase	Not determined	[50,51]
		GH145 α-l-rhamnosidase		
Xylan	1,3-β-d-xylose	GH10 endo-1,3-β-xylanase,	Not determined	[52]
		GH11 endo-β-1,3-xylanase		
		GH43 β-1,3-xylosidase		

PL, Polysaccharide Lyase family; GH, Glycoside Hydrolase family. Potential glycolytic bacteria were identified using the Carbohydrate-Active enZYmes Database [28].

**Table 4:** In vitro fermentation of brown seaweeds with human faecal inoculum.

Seaweed	Substrate	Dose	Use of a Simulated In Vitro Digestion Before Fermentation?	Experimental Parameters	Microbial Enumeration	Microbial Changes	Metabolomics Analysis Technique	Metabolite Changes	Reference
*Ecklonia radiata*	Crude fraction (CF) Phlorotannin-enriched fraction (PF) Low-molecular weight polysaccharide fraction (LPF) High-molecular weight polysaccharide fraction (HPF)	1.5% (*w*/*v*)	Yes CF = 71.5% digestible PF = 87.3% digestible LPF = 86.1% digestible HPF = non-digestible	10% (*w*/*v*) pooled inoculum (n = 3) 24 h	qPCR	↑*Bifidobacterium*↑*Lactobacillus* *(LPF)* ↑*F. prausnitzii*↑*C. coccoides* ↑*Firmicutes* (CF, LPF) ↑*Bacteroidetes*↑*E. coli* (CF, PF, LPF, HPF) ↓*Enterococcus* (CF, PF)	GC-FID	↑Acetate (CF) ↑Propionate (CF, LPF, HPF) ↑ Butyrate (CF, LPF, HPF) ↑ Total SCFA (CF, LPF, HPF)	[56]
*Ecklonia radiata*	Water extract (WE) Acid extract (AE) Celluclast enzyme extract (CEE) Alcalase enzyme extract (AEE) Free sugar fraction (FF) Polysaccharide fraction (PF) Seaweed residue (SR) Seaweed powder (SP)	1.5% (*w*/*v*)	No–digestibility unknown	10% (*w*/*v*) pooled inoculum (n = 3) 24 h	qPCR	= *F. prausnitzii = C. leptum* *= R. bromii* ↑ Total bacteria (CEE, AEE, WE, FF) ↑ *Bifidobacterium* ↑ *Bacteroidetes* ↑ *Lactobacillus* ↑ *C. coccoides* (CEE) ↑ *E. coli* ↑ *Enterocccus* (WE, AE, CEE, AEE, FF, PF, SP)	GC-FID	↑ Acetate ↑ Propionate ↑ Butyrate (WE, AE, CEE, AEE, FF, PF, SP) ↑ Total SCFA	[57]
*Sargassum muticum*	*Sargassum muticum* Alcalase enzyme extract (SAE)	1% (*w*/*v*)	Yes–non-digestible (% digestible undisclosed)	10% (*w*/*v*) single inoculum 24 h	FISH	= *Bifidobacterium* *= Lactobacillus* *= Clostridium histolticum* ↑ *Bacteroides/Prevotella* ↓ *C.coccoides/E.rectale*	HPLC	↑ Total SCFA	[99]
*Sargassum thunbergii*	Polysaccharide extract	0.3% (*w*/*v*)	No–digestibility unknown	20% (*w*/*v*) pooled inoculum (n = 3) 24 h	16S rRNA NGS	↑ Bacteroidetes ↑Bacteroidetes:Firmicutes ratio ↑ *Bifidobacterium* ↑ *Roseburia* ↑ *Parasutterella* ↑ *Fusicatenibacter* ↑ *Coprococcus* ↑ *Fecalibacterium*	GC-MS	↑ Acetate ↑ Propionate ↑ Butyrate ↑ Valerate ↑ Total SCFA	[55]
*-*	Alginate	5% (*w*/*v*)	No–digestibility unknown	10% (*w*/*v*) single inoculum 72 h	16S rRNA DGGE 16S rRNA NGS	↑ *Bacteroides*	GC-FID	↑ Propionate ↑ Butyrate ↑ Total SCFA	[33]
-	Alginate (A) Mannuronic acid oligosaccharides (MO) Guluronic acid oligosaccharides (GO) Propylene glycol alginate sodium sulphate (PSS)	5 g/L (A) 8 g/L (MO, GO, PSS)	No–digestibility unknown	10% (*w*/*v*) single inoculum 48 h	16S rRNA DGGE	Detection of *Bacteroides xylanisolvens, Clostridium clostridioforme/Clostridium symbiosum, Bacteroides finegoldii, Shigella flexneri/E.coli, E.fergusonii,* and *Bacteroides ovatus*	HPLC	A, MO, GO: ↑ Acetate ↑ Propionate ↑ Butyrate ↑ Total SCFA	[36]
*Ascophyllum nodosum*	Sulphated polysaccharide extract	9 mg/mL	Yes–non-digestible (% digestible undisclosed)	10% (*w*/*v*) pooled inoculum (n = 4) 24 h	16S rRNA NGS	↑ *Bacteroides* ↑ *Phascolarctobacterium* ↑ *Oscillospira* ↑ *Fecalibacterium*	GC-FID	↑ Acetate ↑ Propionate ↑ Butyrate ↑ Total SCFA	[100]
*Laminaria digitata*	Crude polysaccharide extract (CE) Depolymerised crude polysaccharide extract (DE)	1% (*w*/*v*)	Yes–non-digestible (% digestible undisclosed)	20% (*w*/*v*) pooled inoculum (n = 3) 48 h	16S rRNA NGS	↑*Parabacteroides* (CE, DE) ↑ *Fibrobacter* (CE) ↓ *Streptococcus* ↓ *Ruminococcus* ↑ *Lachnospiraceae* UC (DE) ↓ *Peptostreptococcaceae* IS (DE) ↑ *Dialister* (CE, DE) ↑ γ B38UC (CE)	GC-FID	↑ Acetate (CE, DE) ↑ Propionate (CE, DE) ↑ Butyrate (CE, DE) ↑ Total SCFA (CE, DE)	[101]
-	Laminarin	1% (*w*/*v*)	No–digestibility unknown	10% (*w*/*v*) pooled inoculum (n = 5) 24 h	qPCR	↑ *Bifidobacterium* ↑ *Bacteroides*	HPLC	↑ Acetate ↑ Propionate ↑ Total SCFA	[79]

qPCR, Quantitative PCR; GC-FID, Gas Chromatography with Flame Ionisation Detector; FISH, Flourescence in situ Hybridisation; 16S rRNA NGS, 16S rRNA Next Generation Sequencing; HPLC, High Performance Liquid Chromaography; GC-MS, Gas Chromatography-Mass Spectrometry; 16S rRNA DGGE, 16S rRNA Denaturing Gradient Gel Electrophoresis; 16S rRNA NGS, 16S rRNA Next Generation Sequencing; GC-FID, Gas Chromatography with Flame Ionisation Detector; HPLC, High Performance Liquid Chromaography; 16S rRNA NGS, 16S rRNA Next Generation Sequencing; qPCR, Quantitative PCR; GC-FID, Gas Chromatography with Flame Ionisation Detector; HPLC, High Performance Liquid Chromatography; SCFA, Short Chain Fatty Acid; =, no statistical difference compared to the control; ↑, significant increase compared to the control; ↓ significant decrease compared to the control. Microbial and metabolite changes with abbreviations in parenthesis indicate the substrate(s) which exerted the effect. If no abbreviations in parenthesis are presented, then all of the seaweed substrates tested exerted the effect.

**Table 5:** In vitro fermentation of red seaweeds with human faecal inoculum.

Seaweed	Substrate	Dose	Use of a Simulated in vitro Digestion Before Fermentation?	Experimental Parameters	Microbial Enumeration	Microbial Changes	Metabolomics Analysis Technique	Metabolite Changes	Reference
*Kappaphycus alvarezii*	Whole Seaweed (WS)	1% (*w*/*v*)	Yes–non-digestible (% digestible undisclosed)	10% (*w*/*v*) single inoculum 24 h	FISH	↑ *Bifidobacterium* ↓ *Clostridium coccoides/* *Eubacterium rectale*	HPLC	↑ Total SCFA	[13]
*Osmundea pinnatifida*	*Osmundea pinnatifida* Viscozyme extract (OVE)	1% (*w*/*v*)	Yes–non-digestible (% digestible undisclosed)	10% (*w*/*v*) single inoculum 24 h	FISH	= *Bifidobacterium* *= Lactobacillus* *= Clostridium histolticum*	HPLC	↑Total SCFA	[99]
*Gracilaria rubra*	Polysaccharide extract (PE)	1% (*w*/*v*)	Yes–non-digestible (% digestible undisclosed)	10% (*w*/*v*) pooled inoculum (n = 4) 24 h	16S rRNA NGS	↑*Bacteroides* ↑*Prevotella* ↑*Phascolarctobacterium* ↓Firmicutes:Bacteroidetes	GC-FID	↑ Acetate ↑ Propionate ↑ Isobutyrate ↑ Total SCFA	[117]
-	Porphyran	1% (*w*/*v*)	No–digestibility unknown	10% (*w*/*v*) pooled inoculum (n = 5) 24 h	qPCR	↑ *Bifidobacterium* ↑ *Bacteroides*	HPLC	= Acetate = Propionate = Butyrate = Total SCFA	[79]

FISH, Flourescence in situ Hybridisation; 16S rRNA NGS, 16S rRNA Next Generation Sequencing; qPCR, Quantitative PCR; GC-FID, Gas Chromatography with Flame Ionisation Detector; HPLC, High Performance Liquid Chromatography; SCFA, Short Chain Fatty Acid; =, no statistical difference compared to the control; ↑, significant increase compared to the control; ↓ significant decrease compared to the control. Microbial and metabolite changes with abbreviations in parenthesis indicate the substrate(s) which exerted the effect. If no abbreviations in parenthesis are presented, then all of the seaweed substrates tested exerted the effect.

**Table 6:** In vitro fermentation of green seaweeds with human faecal inoculum.

Seaweed	Substrate	Dose	Use of a Simulated in vitro Digestion Before Fermentation?	Experimental Parameters	Microbial Enumeration	Microbial Changes	Metabolomics Analysis Technique	Metabolite Changes	Reference
*Enteromorpha prolifera*	Polysaccharide extract (PE)	0.2 g in 9.5 mL 0.8 g in 9.5mL	Yes-non-digestible (% digestible undisclosed)	10.5% (*w*/*v*) pooled inoculum (n = 3) 12, 24, and 48 h	Microbial culture	↑*Enterobacter* (0.2 PE and 0.8 PE at 24 h and 48 h) = *Enterococcus* *= Lactobacillus* *= Bifidobacterium*	GC-FID	= Acetate = Butyrate = Lactate	[12]
-	Ulvan	1% (*w*/*v*)	No-digestibility unknown	10% (*w*/*v*) pooled inoculum (n = 5) 24 h	qPCR	↑ *Bifidobacterium* ↑ *Lactobacillus*	HPLC	↑ Acetate ↑ Lactate	[79]

qPCR, Quantitative PCR; GC-FID, Gas Chromatography with Flame Ionisation Detector; HPLC, High Performance Liquid Chromatography; =, no statistical difference compared to the control; ↑, significant increase compared to the control; ↓ significant decrease compared to the control. Microbial and metabolite changes with abbreviations in parenthesis indicate the substrate(s) which exerted the effect. If no abbreviations in parenthesis are presented, then all of the seaweed substrates tested exerted the effect.

**Table 7:** Impact of seaweeds on the rodent gut microbiota.

Animal	Substrate	Dose	Duration	Biological Sample	Microbial Changes	Metabolite Changes	Reference
30 Male Sprague-Dawley Rats	*Chondrus crispus* Whole Seaweed (WS)	0.5% (*w*/*w*) 2.5% (*w*/*w*)	21 days	Faeces	↑*Bifidobacterium*↑*Legionella*↑*Sutterella* ↑*Blautia*↑*Holdemania* ↑*Shewanella*↑*Agarivorans* ↓*Streptococcus* ↑*Bifidobacterium breve* (2.5% WS)	↑ Acetate ↑ Propionate (2.5% WS) ↑ Butyrate ↑ Total SCFA	[15]
24 Male Sprague-Dawley Rats	*Ecklonia radiata* Whole Seaweed (WS) Ecklonia radiata Polysaccharide Fraction (PF)	5% (*w*/*w*) WS 5% (*w*/*w*) PF	7 days	Caecum	↑*F. prausnitzii*↑*E. coli* (PF) ↓ *Enterococcus* (WS) ↓*Lactobacillus* ↓*Bifidobacterium* ↓ Firmicutes:Bacteroidetes	↑ Acetate ↑ Propionate ↑ Butyrate (PF) ↓ Valerate ↓ Hexanoate ↑ Total SCFA ↓ i-Butyrate ↓ i-Valerate ↓ phenol ↓ p-cresol	[129]
18 Male Wistar Rats	Alginate (A) Laminarin (L) Fucoidan (F)	2% (*w*/*w*)	14 days	Caecum	↑*Bacteroides* (*Bacteroides capillosus*) Presence of *Enterorhabdus* (A) ↑ Proteobacteria. Presence of *Lachnospiracea, Parabacteroides* (*Parabacteroides distasonis*) and *Parasutterella* (L) Not fermented (F)	↑ Propionate (L) ↑ Total SCFA (A, L)	[97]
16 Male C57 BL/6 Mice	*Saccorhiza polyschides* extract (BAE)	High fat diet + 5% (*w*/*w*) BAE	8 months	Faeces	↓ Faecal bile salt hydrolase activity	↓ Secondary bile acids	[54]
18 Male Wistar Rats	Alginate (A) Laminarin (L)	2% (*w*/*w*)	14 days	Caecum	↑*Lactobacillus*↑*Porphyromonas*↑*Coprobacillus* ↑*Oscillibacter valencigenes* ↓*Parabacteroides* (L) ↑ *Catabacter honkongensis* ↑ *Stomatobaculum longum* ↓ *Adlercreuzia* (A) ↓ *Helicobacter* (A, L)	↑ Lactic acid (L) = Acetate = Propionate = Butyrate = Total SCFA ↓ Indole	[130]
18 Male C57BL/6 mice	*Ascophyllum nodosum* Fucoidan (FuA) *Laminaria* *japonica* Fucoidan (FuL)	100 mg/kg/day	6 weeks	Caecum	↑*Lactobacillus*↑*Anaeroplasma* ↑*Thalassospira* (FuA) ↑*Ruminococcaceae* ↓ *Alistipes* ↓ Clostridiales ↓ *Akkermansia* (FuL) ↓ *Candidatus ↓* *Arthromitus* ↓ *Peptococcus* ↓ *Lachnospiraceae Incertae Sedis* (FuA, FuL)	-	[96]
15 Male Wistar rats	*Ascophyllum nodosum* seaweed crude polysaccharide (SCP) SCP *Lactobacillus plantarum* hydrolysate (SCPH Lp) SCP *Enterococcus fecalis* hydrolysate (SCPH Ef) Alginate (A) Hydrolysed Alginate (HA)	0.2 g per 180 –200 g rat weight	4 days	Faeces	-	↑ Acetate (HA > A > SCPH Lp > SCPH Ef) ↑ Propionate (HA = A = SCPH Lp = SCPH Ef) ↑ Butyrate (HA = A = SCPH Lp = SCPH Ef) (relative to day zero)	[131]
32 Female Kunming mice	*Enteromorpha prolifera* (EP) *Enteromorpha* polysaccharide extract (PEP)	1:5 (*w*/*w*)	7 days	Faeces	↑Alpha diversity (EP) ↑*Bacteroidales S24-7* (EP) ↑ *Prevotellaceae* (PEP) ↑ Firmicutes ↑Actinobacteria (EP, PEP) ↓ Bacteroidetes ↓ Proteobacteria (EP, PEP)	-	[119]

SCFA, Short Chain Fatty Acid; =, no statistical difference compared to the control; ↑, significant increase compared to the control; ↓ significant decrease compared to the control. Microbial and metabolite changes with abbreviations in parenthesis indicate the substrate(s) which exerted the effect. If no abbreviations in parenthesis are presented, then all of the seaweed substrates tested exerted the effect.

**Table 8:** Impact of seaweeds on the porcine gut microbiota.

Animal	Seaweed Component	Dose	Duration	Biological Sample	Microbial Changes	Metabolite Changes	Reference
20 pregnant gilts and 48 piglets	Laminarin/Fucoidan Extract	10 g/day	Gestation (day 83) to weaning (day 28)	Faeces (Sow) Colonic digesta (Piglet)	Sows (parturition): ↓ *Enterobacteriaceae* *= Lactobacilli* Piglets (birth, 48h after birth, weaning): = *Enterobacteriaceae = Lactobacilli*	-	[207]
200 pigs	*Ecklonia cava* Whole Seaweed	0.05% (*w*/*w*) 0.1% (*w*/*w*) 0.15% (*w*/*w*)	28 days	Caecum	↑*Lactobacillus* ↓*E. coli* = Total Anaerobes	-	[208]
24 pigs	Laminarin/Fucoidan Extract (SD) Laminarin/Fucoidan Wet Seaweed (WS)	5.37 Kg/tonne SD 26.3 Kg/tonne WS	21 days	Ileum Caecum Colon	*= Bifidobacteria* *= Lactobacillus* *= Enterobacterium* (SD, WS) ↑*Lactobacillus agilis* (colon)	-	[209]
48 pigs	Laminarin Extract	300 ppm	32 days	Faeces	↑ *Lactobacillus* *= Bifidobacteria*	= Acetate ↓ Propionate = Butyrate = Valerate = i-Butyrate = i-Valerate	[210]
48 pigs	β-glucan	250 g/tonne 150 g/tonne	29 days	Ileum Caecum Proximal Colon Distal Colon	= *Lactobacilli* = *Bifidobacteria.* ↑ *Lactobacillus* diversity	-	[211]
168 pigs	Laminarin (L) Fucoidan (F)	240 mg/kg F 150 mg/kg L 300 mg/kg L 150 mg/kg L and 240 mg/kg F 300 mg/kg L and 240 mg/kg F	35 days	Faeces	= *E. coli* = *Bifidobacteria* ↑ *Lactobacilli*	= Acetate = Propionate = Butyrate = Valerate = i-Butyrate = i-Valerate = Total SCFA	[212]
9 pigs	Alginate	5.14% (*w*/*w*)	84 days	Faeces	= Diversity ↑ Unclassified F16 family ↓ *Clostridiaceae* ↓ Unclassified RF39 (Mollicutes) ↑ *Ruminococcus* ↑ *Roseburia* ↑ unclassified *F16* genus (TM7) ↑ *Lachnospira* ↓ *Blautia*	-	[199]

=, no statistical difference compared to the control; ↑, significant increase compared to the control; ↓ significant decrease compared to the control. Microbial and metabolite changes with abbreviations in parenthesis indicate the substrate(s) which exerted the effect. If no abbreviations in parenthesis are presented, then all of the seaweed substrates tested exerted the effect.

**Table 9:** Impact of seaweeds on the hen gut microbiota.

Animal	Seaweed Component	Dose	Duration	Biological Sample	Microbial Changes	Metabolite Changes	Reference
160 laying hens	*Chondrus crispus* Whole Seaweed (CC) *Sarcodiotheca gaudichaudii* Whole Seaweed (SG)	0.5% (*w*/*w*) 1% (*w*/*w*) 2% (*w*/*w*)	30 days	Ileum Caecal digesta	↑*Bifidobacterium longum* (CC2, SG1, SG2) ↑*Streptococcus salivarius* (CC1, CC2, SG2) ↓*Clostridium perfringen*s (CC1, CC2, SG1, SG2) ↓*Lactobacillus acidophilus* (CC1, CC2)	↑ Acetate (CC1, SG1) ↑ Propionate (CC2) ↑ Butyrate (SC2)	[200]
96 laying hens	*Chondrus crispus* Whole Seaweed (CC) *Sarcodiotheca gaudichaudii* Whole Seaweed (SG)	Control diet + 2% (*w*/*w*) seaweed Control diet + 4% (*w*/*w*) seaweed	28 days	Caecum	↑*Lactobacillus acidophilus* (CC4) ↓*Bifidobacterium longum* (SG2, SG4, CC4) ↓ *Streptococcus salivarius* (SG2, SG4, CC2, CC4) ↑ Bacteroidetes (SG4, CC2, CC4)	↑ Propionate (CC4)	[201]

=, no statistical difference compared to the control; ↑, significant increase compared to the control; ↓ significant decrease compared to the control. Microbial and metabolite changes with abbreviations in parenthesis indicate the substrate(s) which exerted the effect. If no abbreviations in parenthesis are presented, then all of the seaweed substrates tested exerted the effect.

**Table 10:** In vitro fermentation of seaweeds with cow rumen inoculum.

Seaweed	Substrate	Experimental Parameters	Dose (*w*/*v*)	Microbial Enumeration	Microbial Changes	Metabolomics Analysis Technique	Metabolite Changes	Reference
*Ascophyllum nodosum* (AN) *Laminaria digitata* (LD)	Whole Seaweed	50% pooled inoculum (n = 4) 24 h	0.5 g/L 1 g/L 2 g/L	-	-	GC-FID	↑Propionate ↑Butyrate (LD) ↓ BCFA ↓Methane	[213]
*Asparagopsis taxiformis*	Whole Seaweed	20% pooled inoculum (n = 4) 72 h	0.5% 1% 2% 5% 10%	-	-	GC-FID	↓ Total gas production ↓ Methane ↓ Acetate ↑ Propionate ↑ Butyrate (2%, 10%) ↓ Total SCFA (5%, 10%)	[214]
*Ulva sp.* *Laminaria ochroleuca* *Saccharina latissima* *Gigartina sp.* *Gracilaria vermiculophylla*	Whole Seaweed	20% pooled inoculum (n = 2) 24 h	25%	-	-	GC-FID	↓ Methane	[215]
Brown seaweed by-products (BSB)	-	50% (*v*/*v*) single inoculum 0, 3, 6, 9, 12, and 24 h	2% 4%	-	-	GC-FID	↓ Ammonia (3, 9, 12 and 24 h) ↓ Total SCFA (24 h)	[216]

GC-FID, Gas Chromatography; =, no statistical difference compared to the control; ↑, significant increase compared to the control; ↓ significant decrease compared to the control. Microbial and metabolite changes with abbreviations in parenthesis indicate the substrate(s) which exerted the effect. If no abbreviations in parenthesis are presented, then all of the seaweed substrates tested exerted the effect.

**Table 11:** Impact of seaweeds on the sheep rumen microbiota.

Seaweed	Dose	Experimental Parameters	Microbial Enumeration	Microbial Changes	Metabolomics Analysis Technique	Metabolite Changes	Reference
*Asparagopsis taxiformis* Whole Seaweed	2%	in vitro batch culture fermentation 20% (*v*/*v*) pooled sheep rumen fluid inoculum (n = 4) 48 and 72 h	16S rRNA NGS qPCR	↓Methanogens ↓Bacteroidetes/Firmicutes ratio ↓ mcrA gene expression	GC-MS	↓ Total Gas ↓ Methane ↑ Hydrogen	[203]
*Ascophyllum nodosum* Whole Seaweed	1% 3% 5%	Rams (n = 8) 21 days *ad libitum*	16S rRNA NGS	↓ undefined *TM7-1* ↓undefined *Coriobacteriaceae* ↓*Roseburia* ↓*Coprococcus* ↓*Prevotella copri* ↑*Blautia producta* ↑ *Entodinium species 1* ↑*Veillonellaceae*	GC-FID	Dose dependent: ↑ Acetate ↓ Propionate ↓ Butyrate PICRUSt: ↑Butanoate metabolism ↑ Fatty acid metabolism ↓Glycerophospholipid metabolism	[204]

16S rRNA NGS, 16S rRNA Next Generation Sequencing; qPCR, Quantitative PCR; GC-FID, Gas Chromatography; =, no statistical difference compared to the control; ↑, significant increase compared to the control; ↓ significant decrease compared to the control. Microbial and metabolite changes with abbreviations in parenthesis indicate the substrate(s) which exerted the effect. If no abbreviations in parenthesis are presented, then all of the seaweed substrates tested exerted the effect.

## References

[1] Brown E.S., Allsopp P.J., Magee P.J., Gill C.I., Nitecki S., Strain C.R., McSorley E.M. (2014). Seaweed and human health. Nutr. Rev..

[2] Cherry P., O’Hara C., Magee P.J., McSorley E.M., Allsopp P.J. (2019). Risks and benefits of consuming edible seaweeds. Nutr. Rev..

[3] De Jesus Raposo M.F., de Morais A.M., de Morais R.M. (2016). Emergent sources of prebiotics: Seaweeds and microalgae. Mar. Drugs.

[4] Thursby E., Juge N. (2017). Introduction to the human gut microbiota. Biochem. J..

[5] Schippa S., Conte M.P. (2014). Dysbiotic events in gut microbiota: Impact on human health. Nutrients.

[6] Gibson G.R., Hutkins R., Sanders M.E., Prescott S.L., Reimer R.A., Salminen S.J., Scott K., Stanton C., Swanson K.S., Cani P.D. (2017). Expert consensus document: The International Scientific Association for Probiotics and Prebiotics (ISAPP) consensus statement on the definition and scope of prebiotics. Nat. Rev. Gastroenterol. Hepatol..

[7] Arnold J.W., Roach J., Azcarate-Peril M.A. (2016). Emerging technologies for gut microbiome research. Trends Microbiol..

[8] O’Sullivan L., Murphy B., McLoughlin P., Duggan P., Lawlor P.G., Hughes H., Gardiner G.E. (2010). Prebiotics from marine macroalgae for human and animal health applications. Mar. Drugs.

[9] Zaporozhets T.S., Besednova N.N., Kuznetsova T.A., Zvyagintseva T.N., Makarenkova I.D., Kryzhanovsky S.P., Melnikov V.G. (2014). The prebiotic potential of polysaccharides and extracts of seaweeds. Russ. J. Mar. Biol..

[10] Devillé C., Damas J., Forget P., Dandrifosse G., Peulen O. (2004). Laminarin in the dietary fibre concept. J. Sci. Food Agric..

[11] Ramnani P., Chitarrari R., Tuohy K., Grant J., Hotchkiss S., Philp K., Campbell R., Gill C., Rowland I. (2012). In vitro fermentation and prebiotic potential of novel low molecular weight polysaccharides derived from agar and alginate seaweeds. Anaerobe.

[12] Kong Q., Dong S., Gao J., Jiang C. (2016). In vitro fermentation of sulfated polysaccharides from *E. prolifera* and *L. japonica* by human fecal microbiota. Int. J. Biol. Macromol..

[13] Bajury D.M., Rawi M.H., Sazali I.H., Abdullah A., Sarbini S.R. (2017). Prebiotic evaluation of red seaweed (*Kappaphycus alvarezii*) using in vitro colon model. Int. J. Food Sci. Nutr..

[14] Devillé C., Gharbi M., Dandrifosse G., Peulen O. (2007). Study on the effects of laminarin, a polysaccharide from seaweed, on gut characteristics. J. Sci. Food Agric..

[15] Liu J., Kandasamy S., Zhang J., Kirby C.W., Karakach T., Hafting J., Critchley A.T., Evans F., Prithiviraj B.J.B.C., Medicine A. (2015). Prebiotic effects of diet supplemented with the cultivated red seaweed Chondrus crispus or with fructo-oligo-saccharide on host immunity, colonic microbiota and gut microbial metabolites. BMC Complement. Altern. Med..

[16] Rose D.J., Keshavarzian A., Patterson J.A., Venkatachalam M., Gillevet P., Hamaker B.R. (2009). Starch-entrapped microspheres extend in vitro fecal fermentation, increase butyrate production, and influence microbiota pattern. Mol. Nutr. Food Res..

[17] Timm D.A., Stewart M.L., Hospattankar A., Slavin J.L. (2010). Wheat dextrin, psyllium, and inulin produce distinct fermentation patterns, gas volumes, and short-chain fatty acid profiles in vitro. J. Med. Food.

[18] Belenguer A., Duncan S.H., Calder A.G., Holtrop G., Louis P., Lobley G.E., Flint H.J. (2006). Two routes of metabolic cross-feeding between Bifidobacterium adolescentis and butyrate-producing anaerobes from the human gut. Appl. Environ. Microbiol..

[19] Macfarlane G.T., Macfarlane S. (2012). Bacteria, colonic fermentation, and gastrointestinal health. J. AOAC Int..

[20] Ríos-Covián D., Ruas-Madiedo P., Margolles A., Gueimonde M., de los Reyes-Gavilán C.G., Salazar N. (2016). Intestinal short chain fatty acids and their link with diet and human health. Front. Microbiol..

[21] Byrne C.S., Chambers E.S., Morrison D.J., Frost G. (2015). The role of short chain fatty acids in appetite regulation and energy homeostasis. Int. J. Obes. (2005).

[22] Gunness P., Gidley M.J. (2010). Mechanisms underlying the cholesterol-lowering properties of soluble dietary fibre polysaccharides. Food Funct..

[23] Den Besten G., van Eunen K., Groen A.K., Venema K., Reijngoud D.-J., Bakker B.M. (2013). The role of short-chain fatty acids in the interplay between diet, gut microbiota, and host energy metabolism. J. Lipid Res..

[24] Tasse L., Bercovici J., Pizzut-Serin S., Robe P., Tap J., Klopp C., Cantarel B.L., Coutinho P.M., Henrissat B., Leclerc M. (2010). Functional metagenomics to mine the human gut microbiome for dietary fiber catabolic enzymes. Genome Res..

[25] El Kaoutari A., Armougom F., Leroy Q., Vialettes B., Million M., Raoult D., Henrissat B. (2013). Development and validation of a microarray for the investigation of the CAZymes encoded by the human gut microbiome. PLoS ONE.

[26] Ndeh D., Gilbert H.J. (2018). Biochemistry of complex glycan depolymerisation by the human gut microbiota. FEMS Microbiol. Rev..

[27] Cecchini D.A., Laville E., Laguerre S., Robe P., Leclerc M., Doré J., Henrissat B., Remaud-Siméon M., Monsan P., Potocki-Véronèse G. (2013). Functional metagenomics reveals novel pathways of prebiotic breakdown by human gut bacteria. PLoS ONE.

[28] Cantarel B.L., Coutinho P.M., Rancurel C., Bernard T., Lombard V., Henrissat B. (2009). The Carbohydrate-Active EnZymes database (CAZy): An expert resource for Glycogenomics. Nucleic Acids Res..

[29] Benitez-Paez A., Gomez Del Pulgar E.M., Sanz Y. (2017). the glycolytic versatility of bacteroides uniformis CECT 7771 and its genome response to oligo and polysaccharides. Front. Cell. Infect. Microbiol..

[30] Brodkorb A., Egger L., Alminger M., Alvito P., Assunção R., Ballance S., Bohn T., Bourlieu-Lacanal C., Boutrou R., Carrière F. (2019). INFOGEST static in vitro simulation of gastrointestinal food digestion. Nat. Protoc..

[31] Thomas F., Barbeyron T., Tonon T., Genicot S., Czjzek M., Michel G. (2012). Characterization of the first alginolytic operons in a marine bacterium: From their emergence in marine Flavobacteriia to their independent transfers to marine Proteobacteria and human gut Bacteroides. Environ. Microbiol..

[32] Brownlee I.A., Allen A., Pearson J.P., Dettmar P.W., Havler M.E., Atherton M.R., Onsoyen E. (2005). Alginate as a source of dietary fiber. Crit. Rev. Food Sci. Nutr..

[33] Bai S., Chen H., Zhu L., Liu W., Yu H.D., Wang X., Yin Y. (2017). Comparative study on the in vitro effects of Pseudomonas aeruginosa and seaweed alginates on human gut microbiota. PLoS ONE.

[34] Li M., Shang Q., Li G., Wang X., Yu G. (2017). Degradation of marine algae-derived carbohydrates by bacteroidetes isolated from human gut microbiota. Mar. Drugs.

[35] Mathieu S., Touvrey-Loiodice M., Poulet L., Drouillard S., Vincentelli R., Henrissat B., Skjåk-Bræk G., Helbert W. (2018). Ancient acquisition of “alginate utilization loci” by human gut microbiota. Sci. Rep..

[36] Li M., Li G., Shang Q., Chen X., Liu W., Pi X., Zhu L., Yin Y., Yu G., Wang X. (2016). In vitro fermentation of alginate and its derivatives by human gut microbiota. Anaerobe.

[37] Zhang J.J., Zhang Q.B., Wang J., Shi X.L., Zhang Z.S. (2009). Analysis of the monosaccharide composition of fucoidan by precolumn derivation HPLC. Chin. J. Oceanol. Limnol..

[38] Salyers A.A., Palmer J.K., Wilkins T.D. (1977). Laminarinase (beta-glucanase) activity in Bacteroides from the human colon. Appl. Environ. Microbiol..

[39] Dousip A., Matanjun P., Sulaiman M.R., Tan T.S., Ooi Y.B.H., Lim T.P.J.J.o.A.P. (2014). Effect of seaweed mixture intake on plasma lipid and antioxidant profile of hyperholesterolaemic rats. J. Appl. Phycol..

[40] Lahaye M., Rochas C. (1991). Chemical-structure and physicochemical properties of agar. Hydrobiologia.

[41] Hehemann J.H., Correc G., Barbeyron T., Helbert W., Czjzek M., Michel G. (2010). Transfer of carbohydrate-active enzymes from marine bacteria to Japanese gut microbiota. Nature.

[42] Rebuffet E., Groisillier A., Thompson A., Jeudy A., Barbeyron T., Czjzek M., Michel G. (2011). Discovery and structural characterization of a novel glycosidase family of marine origin. Environ. Microbiol..

[43] Weiner M.L. (2014). Food additive carrageenan: Part II: A critical review of carrageenan in vivo safety studies. Crit. Rev. Toxicol..

[44] Hehemann J.H., Kelly A.G., Pudlo N.A., Martens E.C., Boraston A.B. (2012). Bacteria of the human gut microbiome catabolize red seaweed glycans with carbohydrate-active enzyme updates from extrinsic microbes. Proc. Natl. Acad. Sci. USA.

[45] Zhang Q.B., Qi H.M., Zhao T.T., Deslandes E., Ismaeli N.M., Molloy F., Critchley A.T. (2005). Chemical characteristics of a polysaccharide from Porphyra capensis (Rhodophyta). Carbohydr. Res..

[46] Usov A.I. (2011). Polysaccharides of the Red Algae. Adv. Carbohydr. Chem. Biochem..

[47] Mirande C., Kadlecikova E., Matulova M., Capek P., Bernalier-Donadille A., Forano E., Bera-Maillet C. (2010). Dietary fibre degradation and fermentation by two xylanolytic bacteria Bacteroides xylanisolvens XB1AT and Roseburia intestinalis XB6B4 from the human intestine. J. Appl. Microbiol..

[48] Hong P.Y., Iakiviak M., Dodd D., Zhang M.L., Mackie R.I., Cann I. (2014). Two new xylanases with different substrate specificities from the human gut bacterium bacteroides intestinalis DSM 17393. Appl. Environ. Microbiol..

[49] Despres J., Forano E., Lepercq P., Comtet-Marre S., Jubelin G., Chambon C., Yeoman C.J., Miller M.E.B., Fields C.J., Martens E. (2016). Xylan degradation by the human gut Bacteroides xylanisolvens XB1A(T) involves two distinct gene clusters that are linked at the transcriptional level. BMC Genom..

[50] Munoz-Munoz J., Cartmell A., Terrapon N., Henrissat B., Gilbert H.J. (2017). Unusual active site location and catalytic apparatus in a glycoside hydrolase family. Proc. Natl. Acad. Sci. USA.

[51] Lahaye M., Robic A. (2007). Structure and functional properties of ulvan, a polysaccharide from green seaweeds. Biomacromolecules.

[52] Liang W.-S., Liu T.C., Chang C.-J., Pan C.-L. (2015). Bioactivity of β-1,3-xylan Extracted from Caulerpa lentillifera by Using Escherichia coli ClearColi BL21(DE3)-β-1,3-xylanase XYLII. J. Food Nutr. Res..

[53] Usman A., Khalid S., Usman A., Hussain Z., Wang Y., Zia K.M., Zuber M., Ali M. (2017). Chapter 5—algal polysaccharides, novel application, and outlook. Algae Based Polymers, Blends, and Composites.

[54] Huebbe P., Nikolai S., Schloesser A., Herebian D., Campbell G., Glüer C.-C., Zeyner A., Demetrowitsch T., Schwarz K., Metges C.C. (2017). An extract from the Atlantic brown algae Saccorhiza polyschides counteracts diet-induced obesity in mice via a gut related multi-factorial mechanisms. Oncotarget.

[55] Fu X., Cao C., Ren B., Zhang B., Huang Q., Li C. (2018). Structural characterization and in vitro fermentation of a novel polysaccharide from Sargassum thunbergii and its impact on gut microbiota. Carbohydr. Polym..

[56] Charoensiddhi S., Conlon M.A., Vuaran M.S., Franco C.M.M., Zhang W. (2017). Polysaccharide and phlorotannin-enriched extracts of the brown seaweed Ecklonia radiata influence human gut microbiota and fermentation in vitro. J. Appl. Phycol..

[57] Charoensiddhi S., Conlon M.A., Vuaran M.S., Franco C.M.M., Zhang W. (2016). Impact of extraction processes on prebiotic potential of the brown seaweed Ecklonia radiata by in vitro human gut bacteria fermentation. J. Funct. Foods.

[58] Vera J., Castro J., Gonzalez A., Moenne A. (2011). Seaweed polysaccharides and derived oligosaccharides stimulate defense responses and protection against pathogens in plants. Mar. Drugs.

[59] García-Ríos V., Ríos-Leal E., Robledo D., Freile-Pelegrin Y. (2012). Polysaccharides composition from tropical brown seaweeds. Phycol. Res..

[60] Maeda-Yamamoto M. (2017). Development of functional agricultural products and use of a new health claim system in Japan. Trends Food Sci. Technol..

[61] Jonathan M.C., Bosch G., Schols H.A., Gruppen H. (2013). Separation and identification of individual alginate oligosaccharides in the feces of alginate-fed pigs. J. Agric. Food Chem..

[62] Jonathan M., Souza da Silva C., Bosch G., Schols H., Gruppen H. (2015). In vivo degradation of alginate in the presence and in the absence of resistant starch. Food Chem..

[63] Terada A., Hara H., Mitsuoka T. (1995). Effect of dietary alginate on the fecal microbiota and fecal metabolic activity in humans. Microb. Ecol. Health Dis..

[64] Wang Y., Han F., Hu B., Li J., Yu W. (2006). In vivo prebiotic properties of alginate oligosaccharides prepared through enzymatic hydrolysis of alginate. Nutr. Res..

[65] Han W., Gu J., Cheng Y., Liu H., Li Y., Li F. (2016). Novel Alginate Lyase (Aly5) from a Polysaccharide-degrading marine bacterium, Flammeovirga sp. Strain MY04: Effects of module truncation on biochemical characteristics, alginate degradation patterns, and oligosaccharide-yielding properties. Appl. Environ. Microbiol..

[66] Chen X.-L., Dong S., Xu F., Dong F., Li P.-Y., Zhang X.-Y., Zhou B.-C., Zhang Y.-Z., Xie B.-B. (2016). Characterization of a New Cold-Adapted and Salt-Activated Polysaccharide Lyase Family 7 Alginate Lyase from Pseudoalteromonas sp. SM0524. Front. Microbiol..

[67] Dong Q., Ruan L., Shi H. (2017). Genome sequence of a high agarase-producing strain Flammeovirga sp. SJP92. Stand. Genom. Sci..

[68] Xu F., Dong F., Wang P., Cao H.Y., Li C.Y., Li P.Y., Pang X.H., Zhang Y.Z., Chen X.L. (2017). Novel molecular insights into the catalytic mechanism of marine bacterial alginate lyase AlyGC from polysaccharide lyase family 6. J. Biol. Chem..

[69] Zhu B., Sun Y., Ni F., Ning L., Yao Z. (2018). Characterization of a new endo-type alginate lyase from Vibrio sp. NJU-03. Int. J. Biol. Macromol..

[70] Liu H., Cheng Y., Gu J., Wang Y., Li J., Li F., Han W. (2017). Draft genome sequence of Paenibacillus sp. Strain MY03, a terrestrial bacterium capable of degrading multiple marine-derived polysaccharides. Genome Announc..

[71] Mathieu S., Henrissat B., Labre F., Skjåk-Bræk G., Helbert W. (2016). Functional exploration of the polysaccharide lyase family PL6. PLoS ONE.

[72] Cantarel B.L., Lombard V., Henrissat B. (2012). Complex carbohydrate utilization by the healthy human microbiome. PLoS ONE.

[73] Hehemann J.-H., Boraston A.B., Czjzek M. (2014). A sweet new wave: Structures and mechanisms of enzymes that digest polysaccharides from marine algae. Curr. Opin. Struct. Biol..

[74] Martin M., Barbeyron T., Martin R., Portetelle D., Michel G., Vandenbol M. (2015). The cultivable surface microbiota of the brown alga Ascophyllum nodosum is enriched in macroalgal-polysaccharide-degrading bacteria. Front Microbiol.

[75] Singh R.P., Reddy C.R.K. (2015). Unraveling the functions of the macroalgal microbiome. Front. Microbiol..

[76] Bhattacharya T., Ghosh T.S., Mande S.S. (2015). global profiling of carbohydrate active enzymes in human gut microbiome. PLoS ONE.

[77] Maruyama Y., Itoh T., Kaneko A., Nishitani Y., Mikami B., Hashimoto W., Murata K. (2015). Structure of a Bacterial ABC Transporter Involved in the Import of an Acidic Polysaccharide Alginate. Structure.

[78] Kadam S.U., O’Donnell C.P., Rai D.K., Hossain M.B., Burgess C.M., Walsh D., Tiwari B.K. (2015). Laminarin from Irish Brown Seaweeds Ascophyllum nodosum and Laminaria hyperborea: Ultrasound Assisted Extraction, Characterization and Bioactivity. Mar. Drugs.

[79] Seong H., Bae J.-H., Seo J.S., Kim S.-A., Kim T.-J., Han N.S. (2019). Comparative analysis of prebiotic effects of seaweed polysaccharides laminaran, porphyran, and ulvan using in vitro human fecal fermentation. J. Funct. Foods.

[80] Brownlee I.A., Havler M.E., Dettmar P.W., Allen A., Pearson J.P. (2003). Colonic mucus: Secretion and turnover in relation to dietary fibre intake. Proc. Nutr. Soc..

[81] Desai M.S., Seekatz A.M., Koropatkin N.M., Kamada N., Hickey C.A., Wolter M., Pudlo N.A., Kitamoto S., Terrapon N., Muller A. (2016). A dietary fiber-deprived gut microbiota degrades the colonic mucus barrier and enhances pathogen susceptibility. Cell.

[82] Salyers A.A., Vercellotti J.R., West S.E., Wilkins T.D. (1977). Fermentation of mucin and plant polysaccharides by strains of Bacteroides from the human colon. Appl. Environ. Microbiol..

[83] Salyers A.A., West S.E., Vercellotti J.R., Wilkins T.D. (1977). Fermentation of mucins and plant polysaccharides by anaerobic bacteria from the human colon. Appl. Environ. Microbiol..

[84] Tailford L.E., Crost E.H., Kavanaugh D., Juge N. (2015). Mucin glycan foraging in the human gut microbiome. Front. Genet..

[85] Dao M.C., Everard A., Aron-Wisnewsky J., Sokolovska N., Prifti E., Verger E.O., Kayser B.D., Levenez F., Chilloux J., Hoyles L. (2016). Akkermansia muciniphila and improved metabolic health during a dietary intervention in obesity: Relationship with gut microbiome richness and ecology. Gut.

[86] Yang L., Wang L., Zhu C., Wu J., Yuan Y., Yu L., Xu Y., Xu J., Wang T., Liao Z. (2017). Laminarin counteracts diet-induced obesity associated with glucagon-like peptide-1 secretion. Oncotarget.

[87] Tolhurst G., Heffron H., Lam Y.S., Parker H.E., Habib A.M., Diakogiannaki E., Cameron J., Grosse J., Reimann F., Gribble F.M. (2012). Short-chain fatty acids stimulate glucagon-like peptide-1 secretion via the G-protein-coupled receptor FFAR2. Diabetes.

[88] Everard A., Cani P.D. (2014). Gut microbiota and GLP-1. Rev. Endocr. Metab. Disord..

[89] Dabek M., McCrae S.I., Stevens V.J., Duncan S.H., Louis P. (2008). Distribution of beta-glucosidase and beta-glucuronidase activity and of beta-glucuronidase gene gus in human colonic bacteria. FEMS Microbiol. Ecol..

[90] Gloux K., Berteau O., El oumami H., Béguet F., Leclerc M., Doré J. (2011). A metagenomic β-glucuronidase uncovers a core adaptive function of the human intestinal microbiome. Proc. Natl. Acad. Sci. USA.

[91] Michalska K., Tan K., Li H., Hatzos-Skintges C., Bearden J., Babnigg G., Joachimiak A. (2013). GH1-family 6-P-β-glucosidases from human microbiome lactic acid bacteria. Acta Crystallogr. Sect. D Biol. Crystallogr..

[92] McNulty N.P., Wu M., Erickson A.R., Pan C., Erickson B.K., Martens E.C., Pudlo N.A., Muegge B.D., Henrissat B., Hettich R.L. (2013). Effects of diet on resource utilization by a model human gut microbiota containing Bacteroides cellulosilyticus WH2, a symbiont with an extensive glycobiome. PLoS Biol..

[93] Tamura K., Hemsworth G.R., Déjean G., Rogers T.E., Pudlo N.A., Urs K., Jain N., Davies G.J., Martens E.C., Brumer H. (2017). Molecular mechanism by which prominent human gut bacteroidetes utilize mixed-linkage beta-glucans, major health-promoting cereal polysaccharides. Cell Rep..

[94] Li B., Lu F., Wei X., Zhao R. (2008). Fucoidan: Structure and Bioactivity. Molecules.

[95] Jiao G., Yu G., Zhang J., Ewart H.S. (2011). Chemical structures and bioactivities of sulfated polysaccharides from marine algae. Mar. Drugs.

[96] Shang Q., Shan X., Cai C., Hao J., Li G., Yu G. (2016). Dietary fucoidan modulates the gut microbiota in mice by increasing the abundance of Lactobacillus and Ruminococcaceae. Food Funct..

[97] An C., Yazaki T., Takahashi H., Kuda T., Kimura B. (2013). Diet-induced changes in alginate- and laminaran-fermenting bacterial levels in the caecal contents of rats. J. Funct. Foods.

[98] Collins K.G., Fitzgerald G.F., Stanton C., Ross R.P. (2016). Looking beyond the terrestrial: The potential of seaweed derived bioactives to treat non-communicable diseases. Mar. Drugs.

[99] Rodrigues D., Walton G., Sousa S., Rocha-Santos T.A.P., Duarte A.C., Freitas A.C., Gomes A.M.P. (2016). In vitro fermentation and prebiotic potential of selected extracts from seaweeds and mushrooms. LWT Food Sci. Technol..

[100] Chen L., Xu W., Chen D., Chen G., Liu J., Zeng X., Shao R., Zhu H. (2018). Digestibility of sulfated polysaccharide from the brown seaweed Ascophyllum nodosum and its effect on the human gut microbiota in vitro. Int. J. Biol. Macromol..

[101] Strain C.R., Collins K.C., Naughton V., McSorley E.M., Stanton C., Smyth T.J., Soler-Vila A., Rea M.C., Ross P.R., Cherry P. (2019). Effects of a polysaccharide-rich extract derived from Irish-sourced Laminaria digitata on the composition and metabolic activity of the human gut microbiota using an in vitro colonic model. Eur. J. Nutr..

[102] Hu B., Gong Q., Wang Y., Ma Y., Li J., Yu W. (2006). Prebiotic effects of neoagaro-oligosaccharides prepared by enzymatic hydrolysis of agarose. Anaerobe.

[103] Li M., Li G., Zhu L., Yin Y., Zhao X., Xiang C., Yu G., Wang X. (2014). Isolation and characterization of an agaro-oligosaccharide (AO)-hydrolyzing bacterium from the gut microflora of Chinese individuals. PLoS ONE.

[104] Zhang Q., Li N., Liu X., Zhao Z., Li Z., Xu Z. (2004). The structure of a sulfated galactan from Porphyra haitanensis and its in vivo antioxidant activity. Carbohydr. Res..

[105] Muraoka T., Ishihara K., Oyamada C., Kunitake H., Hirayama I., Kimura T. (2008). Fermentation Properties of Low-Quality Red Alga Susabinori Porphyra yezoensis by Intestinal Bacteria. Biosci. Biotechnol. Biochem..

[106] Rioux L.-E., Turgeon S.L., Beaulieu M. (2009). Effect of season on the composition of bioactive polysaccharides from the brown seaweed Saccharina longicruris. Phytochemistry.

[107] Kravchenko A.O., Byankina Barabanova A.O., Glazunov V.P., Yakovleva I.M., Yermak I.M. (2018). Seasonal variations in a polysaccharide composition of Far Eastern red seaweed Ahnfeltiopsis flabelliformis (Phyllophoraceae). J. Appl. Phycol..

[108] Skriptsova A.V. (2016). Seasonal variations in the fucoidan content of brown algae from Peter the Great Bay, Sea of Japan. Russ. J. Mar. Biol..

[109] Medcalf D.G., Lionel T., Brannon J.H., Scott J.R. (1975). Seasonal variation in the mucilaginous polysaccharides from Ulva lactuca. Bot. Mar..

[110] Shang Q., Sun W., Shan X., Jiang H., Cai C., Hao J., Li G., Yu G. (2017). Carrageenan-induced colitis is associated with decreased population of anti-inflammatory bacterium, Akkermansia muciniphila, in the gut microbiota of C57BL/6J mice. Toxicol. Lett..

[111] Bhattacharyya S., Liu H., Zhang Z., Jam M., Dudeja P.K., Michel G., Linhardt R.J., Tobacman J.K. (2010). Carrageenan-induced innate immune response is modified by enzymes that hydrolyze distinct galactosidic bonds. J. Nutr. Biochem..

[112] Younes M., Aggett P., Aguilar F., Crebelli R., Filipič M., Frutos M.J., Galtier P., Gott D., Gundert-Remy U., Kuhnle K.K. (2018). Re-evaluation of carrageenan (E 407) and processed Eucheuma seaweed (E 407a) as food additives. EFSA J..

[113] Lahaye M., Michel C., Barry J.L. (1993). Chemical, physicochemical and in-vitro fermentation characteristics of dietary fibres from Palmaria palmata (L.) Kuntze. Food Chem..

[114] Childs C.E., Roytio H., Alhoniemi E., Fekete A.A., Forssten S.D., Hudjec N., Lim Y.N., Steger C.J., Yaqoob P., Tuohy K.M. (2014). Xylo-oligosaccharides alone or in synbiotic combination with Bifidobacterium animalis subsp. lactis induce bifidogenesis and modulate markers of immune function in healthy adults: A double-blind, placebo-controlled, randomised, factorial cross-over study. Br. J. Nutr..

[115] Lecerf J.M., Depeint F., Clerc E., Dugenet Y., Niamba C.N., Rhazi L., Cayzeele A., Abdelnour G., Jaruga A., Younes H. (2012). Xylo-oligosaccharide (XOS) in combination with inulin modulates both the intestinal environment and immune status in healthy subjects, while XOS alone only shows prebiotic properties. Br. J. Nutr..

[116] Mirande C., Mosoni P., Bera-Maillet C., Bernalier-Donadille A., Forano E. (2010). Characterization of Xyn10A, a highly active xylanase from the human gut bacterium Bacteroides xylanisolvens XB1A. Appl. Microbiol. Biotechnol..

[117] Di T., Chen G., Sun Y., Ou S., Zeng X., Ye H. (2018). In vitro digestion by saliva, simulated gastric and small intestinal juices and fermentation by human fecal microbiota of sulfated polysaccharides from Gracilaria rubra. J. Funct. Foods.

[118] Andrieux C., Hibert A., Houari A.-M., Bensaada M., Popot F., Szylit O. (1998). Ulva lactuca is poorly fermented but alters bacterial metabolism in rats inoculated with human fecal flora from methane and non-methane producers. J. Sci. Food Agric..

[119] Ren X., Liu L., Gamallat Y., Zhang B., Xin Y. (2017). Enteromorpha and polysaccharides from enteromorpha ameliorate loperamide-induced constipation in mice. Biomed. Pharmacother..

[120] Charoensiddhi S., Conlon M.A., Franco C.M.M., Zhang W. (2017). The development of seaweed-derived bioactive compounds for use as using enzyme technologies. Trends Food Sci. Technol..

[121] Kim W.J., Park J.W., Park J.K., Choi D.J., Park Y.I. (2015). Purification and Characterization of a Fucoidanase (FNase S) from a Marine Bacterium Sphingomonas paucimobilis PF-1. Mar. Drugs.

[122] Coste O., Malta E.-j., López J.C., Fernández-Díaz C. (2015). Production of sulfated oligosaccharides from the seaweed Ulva sp. using a new ulvan-degrading enzymatic bacterial crude extract. Algal Res..

[123] Barbeyron T., Lerat Y., Sassi J.F., Le Panse S., Helbert W., Collen P.N. (2011). Persicivirga ulvanivorans sp. nov., a marine member of the family Flavobacteriaceae that degrades ulvan from green algae. Int. J. Syst. Evol. Microbiol..

[124] Ramos K.R.M., Valdehuesa K.N.G., Nisola G.M., Lee W.K., Chung W.J. (2018). Identification and characterization of a thermostable endolytic beta-agarase Aga2 from a newly isolated marine agarolytic bacteria Cellulophaga omnivescoria W5C. New Biotechnol..

[125] Xie Z., Lin W., Luo J. (2017). Comparative Phenotype and Genome Analysis of Cellvibrio sp. PR1, a Xylanolytic and Agarolytic Bacterium from the Pearl River. BioMed Res. Int..

[126] Cheng Y., Wang D., Gu J., Li J., Liu H., Li F., Han W. (2017). Biochemical characteristics and variable alginate-degrading modes of a novel bifunctional endolytic alginate lyase. Appl. Environ. Microbiol..

[127] Kislitsyn Y.A., Samygina V.R., Dvortsov I.A., Lunina N.A., Kuranova I.P., Velikodvorskaya G.A. (2015). Crystallization and preliminary X-ray diffraction studies of the family 54 carbohydrate-binding module from laminarinase (beta-1,3-glucanase) Lic16A of Clostridium thermocellum. Acta Crystallogr. Sect. F Struct. Biol. Commun..

[128] Xu X.Q., Su B.M., Xie J.S., Li R.K., Yang J., Lin J., Ye X.Y. (2018). Preparation of bioactive neoagaroligosaccharides through hydrolysis of Gracilaria lemaneiformis agar: A comparative study. Food Chem..

[129] Charoensiddhi S., Conlon M.A., Methacanon P., Franco C.M.M., Su P., Zhang W. (2017). Gut health benefits of brown seaweed Ecklonia radiata and its polysaccharides demonstrated in vivo in a rat model. J. Funct. Foods.

[130] Nakata T., Kyoui D., Takahashi H., Kimura B., Kuda T. (2016). Inhibitory effects of laminaran and alginate on production of putrefactive compounds from soy protein by intestinal microbiota in vitro and in rats. Carbohydr. Polym..

[131] Kaewmanee W., Suwannaporn P., Huang T.C., Al-Ghazzewi F., Tester R.F. (2019). In vivo prebiotic properties of Ascophyllum nodosum polysaccharide hydrolysates from lactic acid fermentation. J. Appl. Phycol..

[132] Gomez-Guzman M., Rodriguez-Nogales A., Algieri F., Galvez J. (2018). potential role of seaweed polyphenols in cardiovascular-associated disorders. Mar. Drugs.

[133] Lee D.H., Park M.Y., Shim B.J., Youn H.J., Hwang H.J., Shin H.C., Jeon H.K. (2012). Effects of Ecklonia cava polyphenol in individuals with hypercholesterolemia: A pilot study. J. Med. Food.

[134] Lopes G., Andrade P.B., Valentao P. (2016). Phlorotannins: Towards new pharmacological interventions for diabetes mellitus type 2. Molecules.

[135] Murray M., Dordevic A.L., Ryan L., Bonham M.P. (2018). An emerging trend in functional foods for the prevention of cardiovascular disease and diabetes: Marine algal polyphenols. Crit. Rev. Food Sci. Nutr..

[136] Murugan A.C., Karim M.R., Yusoff M.B., Tan S.H., Asras M.F., Rashid S.S. (2015). New insights into seaweed polyphenols on glucose homeostasis. Pharm. Biol..

[137] Fernando I.P., Kim M., Son K.T., Jeong Y., Jeon Y.J. (2016). Antioxidant activity of marine algal polyphenolic compounds: A mechanistic approach. J. Med. Food.

[138] Eom S.H., Kim Y.M., Kim S.K. (2012). Antimicrobial effect of phlorotannins from marine brown algae. Food Chem. Toxicol. Int. J. Publ. Br. Ind. Biol. Res. Assoc..

[139] Heffernan N., Brunton N.P., FitzGerald R.J., Smyth T.J. (2015). Profiling of the molecular weight and structural isomer abundance of macroalgae-derived phlorotannins. Mar. Drugs.

[140] Melanson J.E., MacKinnon S.L. (2015). Characterization of Phlorotannins from Brown Algae by LC-HRMS. Methods Mol. Biol..

[141] Montero L., Sanchez-Camargo A.P., Garcia-Canas V., Tanniou A., Stiger-Pouvreau V., Russo M., Rastrelli L., Cifuentes A., Herrero M., Ibanez E. (2016). Anti-proliferative activity and chemical characterization by comprehensive two-dimensional liquid chromatography coupled to mass spectrometry of phlorotannins from the brown macroalga Sargassum muticum collected on North-Atlantic coasts. J. Chromatogr. A.

[142] Lewandowska U., Szewczyk K., Hrabec E., Janecka A., Gorlach S. (2013). Overview of metabolism and bioavailability enhancement of polyphenols. J. Agric. Food Chem..

[143] Opara E.I., Chohan M. (2014). culinary herbs and spices: Their bioactive properties, the contribution of polyphenols and the challenges in deducing their true health benefits. Int. J. Mol. Sci..

[144] Clifford M.N. (2004). Diet-derived phenols in plasma and tissues and their implications for health. Planta Med..

[145] Selma M.V., Espín J.C., Tomás-Barberán F.A. (2009). Interaction between phenolics and gut microbiota: Role in human health. J. Agric. Food Chem..

[146] Williamson G., Clifford M.N. (2017). Role of the small intestine, colon and microbiota in determining the metabolic fate of polyphenols. Biochem. Pharmacol..

[147] Espin J.C., Gonzalez-Sarrias A., Tomas-Barberan F.A. (2017). The gut microbiota: A key factor in the therapeutic effects of (poly)phenols. Biochem. Pharmacol..

[148] Krumholz L.R., Bryant M.P. (1986). Eubacterium oxidoreducens sp. nov. requiring H2 or formate to degrade gallate, pyrogallol, phloroglucinol and quercetin. Arch. Microbiol..

[149] Schneider H., Blaut M. (2000). Anaerobic degradation of flavonoids by Eubacterium ramulus. Arch. Microbiol..

[150] Bang S.H., Hyun Y.J., Shim J., Hong S.W., Kim D.H. (2015). Metabolism of rutin and poncirin by human intestinal microbiota and cloning of their metabolizing alpha-l-rhamnosidase from Bifidobacterium dentium. J. Microbiol. Biotechnol..

[151] Amaretti A., Raimondi S., Leonardi A., Quartieri A., Rossi M. (2015). Hydrolysis of the rutinose-conjugates flavonoids rutin and hesperidin by the gut microbiota and bifidobacteria. Nutrients.

[152] Delgado S., Florez A.B., Guadamuro L., Mayo B. (2017). Genetic and biochemical characterization of an oligo-alpha-1,6-glucosidase from Lactobacillus plantarum. Int. J. Food Microbiol..

[153] Corona G., Ji Y., Anegboonlap P., Hotchkiss S., Gill C., Yaqoob P., Spencer J.P.E., Rowland I. (2016). Gastrointestinal modifications and bioavailability of brown seaweed phlorotannins and effects on inflammatory markers. Br. J. Nutr..

[154] Corona G., Coman M.M., Guo Y., Hotchkiss S., Gill C., Yaqoob P., Spencer J.P.E., Rowland I. (2017). Effect of simulated gastrointestinal digestion and fermentation on polyphenolic content and bioactivity of brown seaweed phlorotannin-rich extracts. Mol. Nutr. Food Res..

[155] Sadeghi Ekbatan S., Sleno L., Sabally K., Khairallah J., Azadi B., Rodes L., Prakash S., Donnelly D.J., Kubow S. (2016). Biotransformation of polyphenols in a dynamic multistage gastrointestinal model. Food Chem..

[156] Oliveira A., Pintado M. (2015). Stability of polyphenols and carotenoids in strawberry and peach yoghurt throughout in vitro gastrointestinal digestion. Food Funct..

[157] Brown E.M., Nitecki S., Pereira-Caro G., McDougall G.J., Stewart D., Rowland I., Crozier A., Gill C.I. (2014). Comparison of in vivo and in vitro digestion on polyphenol composition in lingonberries: Potential impact on colonic health. BioFactors.

[158] Dueñas M., Muñoz-González I., Cueva C., Jiménez-Girón A., Sánchez-Patán F., Santos-Buelga C., Moreno-Arribas M.V., Bartolomé B. (2015). A survey of modulation of gut microbiota by dietary polyphenols. BioMed Res. Int..

[159] Tomas-Barberan F.A., Selma M.V., Espin J.C. (2016). Interactions of gut microbiota with dietary polyphenols and consequences to human health. Curr. Opin. Clin. Nutr. Metab. Care.

[160] Duda-Chodak A., Tarko T., Satora P., Sroka P. (2015). Interaction of dietary compounds, especially polyphenols, with the intestinal microbiota: A review. Eur. J. Nutr..

[161] Nunez-Sanchez M.A., Gonzalez-Sarrias A., Romo-Vaquero M., Garcia-Villalba R., Selma M.V., Tomas-Barberan F.A., Garcia-Conesa M.T., Espin J.C. (2015). Dietary phenolics against colorectal cancer--From promising preclinical results to poor translation into clinical trials: Pitfalls and future needs. Mol. Nutr. Food Res..

[162] Ozdal T., Sela D.A., Xiao J., Boyacioglu D., Chen F., Capanoglu E. (2016). The Reciprocal Interactions between Polyphenols and Gut Microbiota and Effects on Bioaccessibility. Nutrients.

[163] Van Duynhoven J., Vaughan E.E., Jacobs D.M., Kemperman R.A., van Velzen E.J., Gross G., Roger L.C., Possemiers S., Smilde A.K., Dore J. (2011). Metabolic fate of polyphenols in the human superorganism. Proc. Natl. Acad. Sci. USA.

[164] Rubio L., Macia A., Motilva M.J. (2014). Impact of various factors on pharmacokinetics of bioactive polyphenols: An overview. Curr. Drug Metab..

[165] Feliciano R.P., Mills C.E., Istas G., Heiss C., Rodriguez-Mateos A. (2017). Absorption, metabolism and excretion of cranberry (Poly)phenols in humans: A dose response study and assessment of inter-individual variability. Nutrients.

[166] Dueñas M., Cueva C., Muñoz-González I., Jiménez-Girón A., Sánchez-Patán F., Santos-Buelga C., Moreno-Arribas M.V., Bartolomé B. (2015). Studies on modulation of gut microbiota by wine polyphenols: From isolated cultures to omic approaches. Antioxidants.

[167] Rajauria G., Foley B., Abu-Ghannam N. (2017). Characterization of dietary fucoxanthin from Himanthalia elongata brown seaweed. Food Res. Int..

[168] Christaki E., Bonos E., Giannenas I., Florou-Paneri P. (2013). Functional properties of carotenoids originating from algae. J. Sci. Food Agric..

[169] Mikami K., Hosokawa M. (2013). Biosynthetic pathway and health benefits of fucoxanthin, an algae-specific xanthophyll in brown seaweeds. Int. J. Mol. Sci..

[170] Lopes-Costa E., Abreu M., Gargiulo D., Rocha E., Ramos A.A. (2017). Anticancer effects of seaweed compounds fucoxanthin and phloroglucinol, alone and in combination with 5-fluorouracil in colon cells. J. Toxicol. Environ. Health.

[171] Maeda H., Tsukui T., Sashima T., Hosokawa M., Miyashita K. (2008). Seaweed carotenoid, fucoxanthin, as a multi-functional nutrient. Asia Pac. J. Clin. Nutr..

[172] Woo M.N., Jeon S.M., Kim H.J., Lee M.K., Shin S.K., Shin Y.C., Park Y.B., Choi M.S. (2010). Fucoxanthin supplementation improves plasma and hepatic lipid metabolism and blood glucose concentration in high-fat fed C57BL/6N mice. Chem. Biol. Interact..

[173] Kulczyński B., Gramza-Michałowska A., Kobus-Cisowska J., Kmiecik D. (2017). The role of carotenoids in the prevention and treatment of cardiovascular disease–Current state of knowledge. J. Funct. Foods.

[174] Bohn T., McDougall G.J., Alegria A., Alminger M., Arrigoni E., Aura A.M., Brito C., Cilla A., El S.N., Karakaya S. (2015). Mind the gap-deficits in our knowledge of aspects impacting the bioavailability of phytochemicals and their metabolites—A position paper focusing on carotenoids and polyphenols. Mol. Nutr. Food Res..

[175] Widjaja-Adhi M.A.K., Lobo G.P., Golczak M., Von Lintig J. (2015). A genetic dissection of intestinal fat-soluble vitamin and carotenoid absorption. Hum. Mol. Genet..

[176] Rein M.J., Renouf M., Cruz-Hernandez C., Actis-Goretta L., Thakkar S.K., da Silva Pinto M. (2013). Bioavailability of bioactive food compounds: A challenging journey to bioefficacy. Br. J. Clin. Pharmacol..

[177] Bohn T., Saura-Calixto F., Pérez-Jiménez J. (2018). Chapter 9 Metabolic Fate of Bioaccessible and Non-bioaccessible Carotenoids. Non-Extractable Polyphenols and Carotenoids: Importance in Human Nutrition and Health.

[178] Lyu Y., Wu L., Wang F., Shen X., Lin D. (2018). Carotenoid supplementation and retinoic acid in immunoglobulin A regulation of the gut microbiota dysbiosis. Exp. Biol. Med..

[179] Kamiloglu S., Capanoglu E., Saura-Calixto F., Pérez-Jiménez J. (2018). Chapter 10 models for studying polyphenols and carotenoids digestion, bioaccessibility and colonic fermentation. Non-Extractable Polyphenols and Carotenoids: Importance in Human Nutrition and Health.

[180] Van Ginneken V.J.T., Helsper J.P.F.G., de Visser W., van Keulen H., Brandenburg W.A. (2011). Polyunsaturated fatty acids in various macroalgal species from north Atlantic and tropical seas. Lipids Health Dis..

[181] Robertson R.C., Guihéneuf F., Bahar B., Schmid M., Stengel D.B., Fitzgerald G.F., Ross R.P., Stanton C. (2015). The Anti-Inflammatory Effect of Algae-Derived Lipid Extracts on Lipopolysaccharide (LPS)-Stimulated Human THP-1 Macrophages. Mar. Drugs.

[182] Costantini L., Molinari R., Farinon B., Merendino N. (2017). Impact of Omega-3 fatty acids on the gut microbiota. Int. J. Mol. Sci..

[183] Menni C., Zierer J., Pallister T., Jackson M.A., Long T., Mohney R.P., Steves C.J., Spector T.D., Valdes A.M. (2017). Omega-3 fatty acids correlate with gut microbiome diversity and production of N-carbamylglutamate in middle aged and elderly women. Sci. Rep..

[184] Robertson R.C., Kaliannan K., Strain C.R., Ross R.P., Stanton C., Kang J.X. (2018). Maternal omega-3 fatty acids regulate offspring obesity through persistent modulation of gut microbiota. Microbiome.

[185] Marco M.L., Heeney D., Binda S., Cifelli C.J., Cotter P.D., Foligne B., Ganzle M., Kort R., Pasin G., Pihlanto A. (2017). Health benefits of fermented foods: Microbiota and beyond. Curr. Opin. Biotechnol..

[186] Chilton S.N., Burton J.P., Reid G. (2015). Inclusion of fermented foods in food guides around the world. Nutrients.

[187] Tamang J.P., Shin D.H., Jung S.J., Chae S.W. (2016). Functional properties of microorganisms in fermented foods. Front. Microbiol..

[188] Wilburn J.R., Ryan E.P., Frias J., Martinez-Villaluenga C., Peñas E. (2017). Chapter 1—Fermented foods in health promotion and disease prevention: An overview. Fermented Foods in Health and Disease Prevention.

[189] Ko S.J., Kim J., Han G., Kim S.K., Kim H.G., Yeo I., Ryu B., Park J.W. (2014). Laminaria japonica combined with probiotics improves intestinal microbiota: A randomized clinical trial. J. Med. Food.

[190] Kang Y.M., Lee B.J., Kim J.I., Nam B.H., Cha J.Y., Kim Y.M., Ahn C.B., Choi J.S., Choi I.S., Je J.Y. (2012). Antioxidant effects of fermented sea tangle (Laminaria japonica) by Lactobacillus brevis BJ20 in individuals with high level of gamma-GT: A randomized, double-blind, and placebo-controlled clinical study. Food Chem. Toxicol. Int. J. Publ. Br. Ind. Biol. Res. Assoc..

[191] Choi J.S., Seo H.J., Lee Y.R., Kwon S.J., Moon S.H., Park S.M., Sohn J.H. (2014). Characteristics and in vitro Anti-diabetic Properties of the Korean Rice Wine, Makgeolli Fermented with Laminaria japonica. Prev. Nutr. Food Sci..

[192] Shobharani P., Nanishankar V.H., Halami P.M., Sachindra N.M. (2014). Antioxidant and anticoagulant activity of polyphenol and polysaccharides from fermented Sargassum sp.. Int. J. Biol. Macromol..

[193] Uchida M., Miyoshi T., Yoshida G., Niwa K., Mori M., Wakabayashi H. (2014). Isolation and characterization of halophilic lactic acid bacteria acting as a starter culture for sauce fermentation of the red alga Nori (Porphyra yezoensis). J. Appl. Microbiol..

[194] Tavares Estevam A.C., Alonso Buriti F.C., de Oliveira T.A., Pereira E.V., Florentino E.R., Porto A.L. (2016). Effect of Aqueous Extract of the Seaweed Gracilaria domingensis on the Physicochemical, Microbiological, and Textural Features of Fermented Milks. J. Food Sci..

[195] Blaszak B.B., Gozdecka G., Shyichuk A. (2018). Carrageenan as a functional additive in the production of cheese and cheese-like products. Acta Sci. Polonorum. Technol. Aliment..

[196] Bixler H.J., Porse H.J. (2011). A decade of change in the seaweed hydrocolloids industry. J. Appl. Phycol..

[197] Makkar H.P.S., Tran G., Heuzé V., Giger-Reverdin S., Lessire M., Lebas F., Ankers P. (2016). Seaweeds for livestock diets: A review. Anim. Feed Sci. Technol..

[198] Overland M., Mydland L.T., Skrede A. (2018). Marine macroalgae as sources of protein and bioactive compounds in feed for monogastric animals. J. Sci. Food Agric..

[199] Umu O.C., Frank J.A., Fangel J.U., Oostindjer M., da Silva C.S., Bolhuis E.J., Bosch G., Willats W.G., Pope P.B., Diep D.B. (2015). Resistant starch diet induces change in the swine microbiome and a predominance of beneficial bacterial populations. Microbiome.

[200] Kulshreshtha G., Rathgeber B., Stratton G., Thomas N., Evans F., Critchley A., Hafting J., Prithiviraj B. (2014). Feed supplementation with red seaweeds, Chondrus crispus and Sarcodiotheca gaudichaudii, affects performance, egg quality, and gut microbiota of layer hens. Poult. Sci..

[201] Kulshreshtha G., Rathgeber B., MacIsaac J., Boulianne M., Brigitte L., Stratton G., Thomas N.A., Critchley A.T., Hafting J., Prithiviraj B. (2017). Feed Supplementation with Red Seaweeds, Chondrus crispus and Sarcodiotheca gaudichaudii, Reduce Salmonella Enteritidis in Laying Hens. Front. Microbiol..

[202] Litten-Brown J.C., Corson A.M., Clarke L. (2010). Porcine models for the metabolic syndrome, digestive and bone disorders: A general overview. Animal.

[203] Machado L., Tomkins N., Magnusson M., Midgley D.J., de Nys R., Rosewarne C.P. (2018). In Vitro Response of Rumen Microbiota to the Antimethanogenic Red Macroalga Asparagopsis taxiformis. Microb. Ecol..

[204] Zhou M., Hünerberg M., Chen Y., Reuter T., McAllister T.A., Evans F., Critchley A.T., Guan L.L. (2018). Air-Dried Brown Seaweed, Ascophyllum nodosum, Alters the Rumen Microbiome in a Manner That Changes Rumen Fermentation Profiles and Lowers the Prevalence of Foodborne Pathogens. mSphere.

[205] Orpin C.G., Greenwood Y., Hall F.J., Paterson I.W. (1985). The rumen microbiology of seaweed digestion in Orkney sheep. J. Appl. Bacteriol..

[206] Williams A.G., Withers S., Sutherland A.D. (2013). The potential of bacteria isolated from ruminal contents of seaweed-eating North Ronaldsay sheep to hydrolyse seaweed components and produce methane by anaerobic digestion in vitro. Microb. Biotechnol..

[207] Heim G., O’Doherty J.V., O’Shea C.J., Doyle D.N., Egan A.M., Thornton K., Sweeney T. (2015). Maternal supplementation of seaweed-derived polysaccharides improves intestinal health and immune status of suckling piglets. J. Nutr. Sci..

[208] Choi Y., Hosseindoust A., Goel A., Lee S., Jha P.K., Kwon I.K., Chae B.-J. (2017). Effects of Ecklonia cava as fucoidan-rich algae on growth performance, nutrient digestibility, intestinal morphology and caecal microflora in weanling pigs. Asian Australas. J. Anim. Sci..

[209] Murphy P., Dal Bello F., O’Doherty J., Arendt E.K., Sweeney T., Coffey A. (2013). The effects of liquid versus spray-dried Laminaria digitata extract on selected bacterial groups in the piglet gastrointestinal tract (GIT) microbiota. Anaerobe.

[210] Heim G., Walsh A.M., Sweeney T., Doyle D.N., O’Shea C.J., Ryan M.T., O’Doherty J.V. (2014). Effect of seaweed-derived laminarin and fucoidan and zinc oxide on gut morphology, nutrient transporters, nutrient digestibility, growth performance and selected microbial populations in weaned pigs. Br. J. Nutr..

[211] Murphy P., Dal Bello F., O’Doherty J., Arendt E.K., Sweeney T., Coffey A. (2013). Analysis of bacterial community shifts in the gastrointestinal tract of pigs fed diets supplemented with β-glucan from Laminaria digitata, Laminaria hyperborea and Saccharomyces cerevisiae. Animal.

[212] Walsh A.M., Sweeney T., O’Shea C.J., Doyle D.N., ’Doherty J.V.O. (2013). Effect of supplementing varying inclusion levels of laminarin and fucoidan on growth performance, digestibility of diet components, selected fecal microbial populations and volatile fatty acid concentrations in weaned pigs. Anim. Feed Sci. Technol..

[213] Belanche A., Ramos-Morales E., Newbold C.J. (2016). In vitro screening of natural feed additives from crustaceans, diatoms, seaweeds and plant extracts to manipulate rumen fermentation. J. Sci. Food Agric..

[214] Kinley R.D., de Nys R., Vucko M.J., Machado L., Tomkins N.W. (2016). The red macroalgae Asparagopsis taxiformis is a potent natural antimethanogenic that reduces methane production during in vitro fermentation with rumen fluid. J. Anim. Prod. Sci..

[215] Maia M.R., Fonseca A.J., Oliveira H.M., Mendonca C., Cabrita A.R. (2016). The potential role of seaweeds in the natural manipulation of rumen fermentation and methane production. Sci. Rep..

[216] Hong Z.S., Kim E.J., Jin Y.C., Lee J.S., Choi Y.J., Lee H.G. (2015). Effects of supplementing brown seaweed by-products in the diet of Holstein cows during transition on ruminal fermentation, growth performance and endocrine responses. Asian Australas. J. Anim. Sci..

